# Effects of Type and Timing of Clinician-Facing AI Support on Patient Trust in Medical Consultations: 2 Vignette Experiments

**DOI:** 10.2196/93172

**Published:** 2026-08-21

**Authors:** Insa Schaffernak, Julia Cecil, Eesha Kokje, Anne-Kathrin Kleine, Amr Saad, Filmon Zemo, Eva Lermer

**Affiliations:** 1 Department of Business Psychology, Technische Hochschule Augsburg, An der Hochschule 1, Augsburg, 86161, Germany; 2 Center for Leadership and People Management, Ludwig-Maximilians-Universität München, Munich, Germany; 3 Department of Ophthalmology, Stadtspital Zürich, Zurich, Switzerland; 4 Spross Research Institute, Stadtspital Zürich, Zurich, Switzerland

**Keywords:** artificial intelligence, clinical decision support systems, patient trust, compliance, patient-physician relationship, decision-making, ophthalmology, vignette studies, medical informatics applications

## Abstract

**Background:**

AI-based clinical decision support systems are increasingly integrated into medical practice, creating hybrid decision-making processes in which physicians and AI systems jointly contribute to clinical judgments. Yet, how different forms of such AI support affect patients’ trust in hybrid medical decisions remains poorly understood.

**Objective:**

This study aimed to examine how the type and the timing of physician AI support influence potential patients’ trust in the medical decisions, perceptions of the hybrid decision-making process, and intentions to follow the medical advice.

**Methods:**

In 2 preregistered vignette-based online experiments, 489 (study 1) and 570 (study 2) members of the general public in Germany imagined 4 medical consultations, in which the physician used no AI support, descriptive AI support (informational or visual assistance), or diagnostic AI support (preliminary diagnostic suggestions). Study 2 additionally manipulated the timing of AI support, namely, whether the physician reviewed AI advice after having made an independent own assessment (sequential decision-making) or not (concurrent decision-making). Participants rated their trust in the medical decisions, trustworthiness of the medical provider, uniqueness neglect, and willingness to follow the medical advice on 7-point Likert scales, with greater values representing stronger agreement. Linear mixed-effects models were used for quantitative analyses. Open-ended responses (N=2607) were analyzed qualitatively to identify recurring themes regarding trust in AI-supported decisions.

**Results:**

In study 1, the physician’s use of diagnostic AI support compared with descriptive AI support produced significantly lower mean ratings of trust in the medical decisions (5.00 vs 5.37; *t*_486_=3.51; *P=*.002) and perceived provider trustworthiness (4.93 vs 5.40; *t*_486_=4.46; *P<*.001), as well as higher mean ratings of perceived uniqueness neglect (3.24 vs 2.93; *t*_486_=2.72; *P=*.02), but no significant differences regarding the willingness to follow the advice (5.42 vs 5.62; *t*_486_=1.74; *P=*.25). Study 2 replicated these results and revealed a significant interaction effect between type and timing of AI support for trust in the medical decisions (*t*_436_=2.71; *P=*.007), perceived provider trustworthiness (*t*_436_=2.78; *P=*.006), and perceived uniqueness neglect (*t*_436_=−2.34; *P=*.02) but not for willingness to follow the advice (*t*_436_=1.81; *P=*.07). Specifically, diagnostic AI support was evaluated less favorably than descriptive AI support when the physician reviewed primary medical information and AI output simultaneously but not when the physician first assessed the primary medical information independently before reviewing the AI output. Qualitative responses showed that participants were concerned that erroneous AI output biases physicians’ judgments and indicated that physician independence could strengthen trust.

**Conclusions:**

The use of AI to support physicians appears more acceptable when used for analytical rather than decisional support, or when physicians’ decisional independence is visibly retained, suggesting that trust in AI-supported medical decisions depends not only on whether physicians use AI support but also on subjective perceptions of how it is used.

## Introduction

### Background

AI-based clinical decision support systems (AI-CDSS) are increasingly integrated into health care, where they are expected to improve clinical decision-making and, ultimately, patient outcomes [1,2]. These systems assist physicians by analyzing complex data, visualizing trends, quantifying biomarkers, or providing diagnostic and treatment suggestions [3,4]. AI-CDSS are especially valuable for image-based clinical decisions, where AI models have demonstrated performance comparable with or exceeding that of human experts across multiple medical specialties [5-7]. At the same time, there is ongoing debate about how to integrate these systems into clinical practice in ways that respect the needs of all stakeholders [8-11].

One of these stakeholder groups are patients, who do not directly interact with clinician-facing AI-CDSS but are affected by the resulting medical decision. Emerging ethical, legal, and regulatory frameworks increasingly emphasize that patients should be informed when AI-CDSS are involved in their care [12-16]. Empirical studies further show that patients expect physicians to disclose AI involvement and to explain how algorithmic recommendations contribute to clinical decisions [17-19]. Such transparency is viewed as essential for maintaining trust, respecting patient autonomy, and supporting shared decision-making [20].

As AI-CDSS become more widely used, patients are increasingly confronted with *hybrid* medical decisions, in which physician and algorithm jointly contribute to the clinical judgment. While these systems promise to improve efficiency and diagnostic accuracy, they also introduce new challenges from the patients’ point of view. For example, hybrid decision-making may create uncertainty about responsibility, physician competence, or the quality of the final decision [21,22]. The use of AI-CDSS therefore has the potential to affect patients’ trust in the decision-making process and associated medical recommendations, which ultimately determines whether the use of AI-CDSS in clinical practice really improves patient outcomes: If patients question the validity of a diagnosis or recommendation, they may be less inclined to follow the medical advice, thereby undermining the potential benefits of even the most accurate systems [23-25]. Therefore, understanding the sociopsychological consequences of hybrid medical decision-making—from the perspective of those affected by such decisions—is crucial for a successful and ethical implementation of AI-CDSS in clinical practice.

### Patients’ Trust in AI-Assisted Medical Decisions

Patients’ trust in the physician and medical decisions is a key determinant of adherence to recommended care [23,25]. Conversely, questioning the validity of a diagnosis or recommendation may lead patients to seek second opinions—adding pressure on already strained health care systems [26]—delay timely care, or even deter them from receiving necessary treatment altogether.

Crucially, a growing body of research suggests that people tend to prefer and follow medical advice from human physicians over advice generated by AI systems [27-32]. Longoni et al [33] proposed that this asymmetry is partly driven by *perceived uniqueness neglect*—the belief that algorithms are less capable than humans of considering a patient’s unique context, needs, or preferences. When it comes to *hybrid* medical decisions, in which AI supports rather than replaces the physician, findings are mixed. Survey and interview studies suggest that many patients are willing to accept AI in a supportive role [34-39]. Experimental vignette studies, however, show inconsistent results. Some studies reported comparable levels of acceptance and perceived trustworthiness for hybrid and physician-only decision-making [33,40], whereas others found reduced perceptions of competence, empathy, or trustworthiness when AI involvement is disclosed [22,41]. Similarly, intentions to follow hybrid recommendations have been reported as lower than for physician-only care in certain patient groups [42]. Some evidence also suggested a decreasing pattern of trust from physician-only to hybrid to AI-only decisions [43]. Together, these inconsistencies could indicate that patient perceptions of hybrid decision-making depend on more than the mere presence of AI. Instead, the specific way in which AI supports the physician may play a crucial role.

### Variation in AI Involvement in Hybrid Medical Decision-Making

In clinical practice, physician-facing AI support can vary both in the *type* of support provided and in the *timing* at which it is used in the clinical decision-making process. Regarding the type of AI support, available AI-CDSS differ substantially in their functionalities [9,44]. Some systems, for example, serve in a clearly assistive role by visualizing biomarkers, highlighting regions of interest, or quantifying physiological changes over time. This type of AI support improves the physicians’ informational basis but still requires them to synthesize the data, weigh contextual factors, and formulate the clinical judgment [45]. We refer to these systems as *descriptive* AI support. In contrast, other systems already provide an integrated output, such as a preliminary diagnosis or treatment recommendations, shifting the AI’s role from informational support to interpretive guidance. These systems may reduce the physician’s cognitive burden but are also associated with some risk of overreliance on AI outputs [46-49]. We refer to these systems as *diagnostic* AI support.

Regarding the timing of AI support, a recent review differentiated between *concurrent* decision-making, where AI outputs are reviewed alongside the primary clinical information (eg, medical imaging), and *sequential* decision-making, where the physician reviews AI output only after assessing the primary clinical information first [9].

Depending on how AI-CDSS are used in medical consultations in terms of the type and timing of support, AI systems assume different roles in the clinical decision-making process. These differences in AI involvement may influence how patients perceive the clinical decision-making process and how much trust they place in the final recommendation. Research on perceptions of algorithmic decision-making outside medicine, for example, suggests that acceptance of AI use depends on the type of task for which it is used, with higher acceptance for data-driven or mechanical tasks than for tasks requiring human judgment or empathy [50,51]. Regarding the timing of AI support, concerns have been raised that concurrent workflows increase the risk of undue reliance on potentially incorrect AI advice compared with sequential workflows, as clinicians may be less likely to form independent assessments [8,52-54]. However, little is known about how variations in the type and timing of AI support influence potential patients’ trust in AI-assisted medical decisions. Filling this gap is crucial to anticipate patients’ experience of the increasingly hybrid nature of medical decision-making.

### Objectives

In response to growing calls for research on the sociopsychological consequences of AI-assisted medical decision-making [21,34,55], we conducted 2 preregistered vignette experiments to examine how different approaches to implementing AI-CDSS in medical consultations influence potential patients’ trust in the resulting medical decisions. Such trust is essential for ensuring that AI-supported care does not lower adherence but actually translates into improved clinical outcomes.

Prior quantitative research on perceptions of AI-supported care has typically focused on a single implementation of AI-CDSS and compared it with physician-only or AI-only decisions. In contrast, we adopt a more nuanced approach by systematically comparing different ways in which AI support can be involved in medical decision-making, varying by the type and timing of AI support. The investigated variations in AI involvement were informed by realistic and currently debated approaches to implementing AI-CDSS in clinical practice [9,44,45]. To our knowledge, this is the first study to systematically examine the combined effects of both the type of AI support and the timing of its use within clinical decision-making, enabling a more nuanced assessment of how distinct features of AI-assisted care influence potential patients’ trust. Additionally, we extend prior work by combining large-scale quantitative analyses with qualitative insights from open-ended responses, enabling a deeper understanding of participants’ reasoning while retaining the strengths of experimental designs.

We investigated the following overarching research questions:

- Research question 1: How does the *type of AI support* influence potential patients’ evaluations of hybrid (ie, AI-assisted) medical decisions?
- Research question 2: How does the *timing of AI support* within the medical decision-making process influence potential patients’ evaluations of hybrid medical decisions?
- Research question 3: How can potential patients’ trust in hybrid medical decisions be improved?

## Methods

### Study Overview

**Figure 1.**
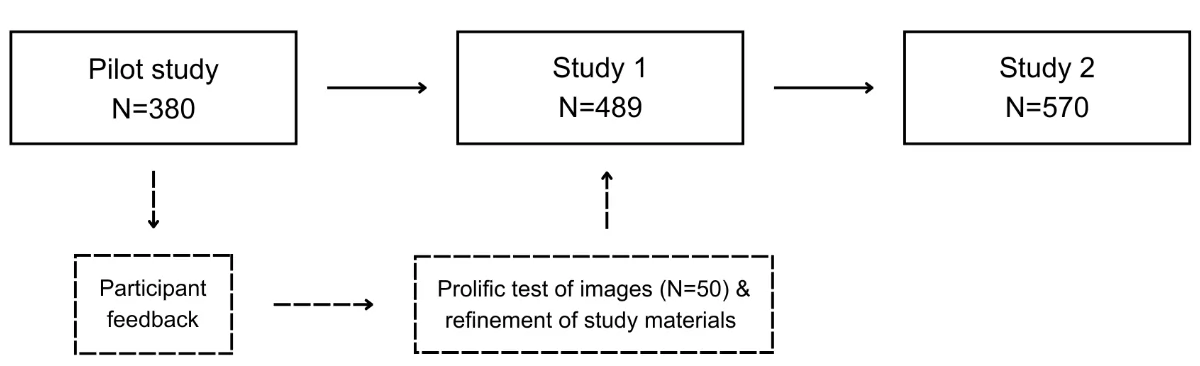
Overview of the research design. Following a pilot study and refinement of the study materials based on participant feedback, we conducted 2 preregistered vignette-based experiments with 489 and 570 participants.

To address the research questions, we conducted a series of online vignette experiments comprising a pilot study followed by 2 large-scale main studies (Figure 1). These studies systematically examined how the type and timing of AI support influence potential (ie, analogue) patients’ trust in the resulting hybrid medical decisions, perceived trustworthiness of the medical provider, perceived uniqueness neglect, and willingness to follow the medical advice.

We adopted a vignette-based experimental approach because it allows for the controlled manipulation of distinct aspects of AI involvement in clinical decision-making, such as support type and timing. Moreover, this approach avoids ethical and practical constraints associated with exposing real patients to experimental variations of AI-supported care while still allowing participants to engage with realistic medical scenarios [56].

Study materials were developed and refined in close collaboration with physicians to ensure clinical plausibility and relevance. Prior to the main data collection, we conducted a pilot study (N=380) to evaluate the clarity and realism of the newly developed vignettes and to obtain preliminary effect size estimates for sample size planning. Participants were invited to provide feedback on how immersion in the vignettes could be improved. Based on their suggestions, we refined the materials by adding greater detail to the clinical encounters and incorporating 3 contextual images per vignette. To select appropriate images, we pretested a pool of images via Prolific (N=50) and selected images with comparable valence and arousal ratings for each vignette. Retinal images were retrieved from publicly available datasets [57-60]. In addition, we further refined the materials by concretizing the manipulation checks and moving demographic questions to the end of the survey. More information about the pilot study and the image pretest can be found in Multimedia Appendix 1.

**Figure 2.**
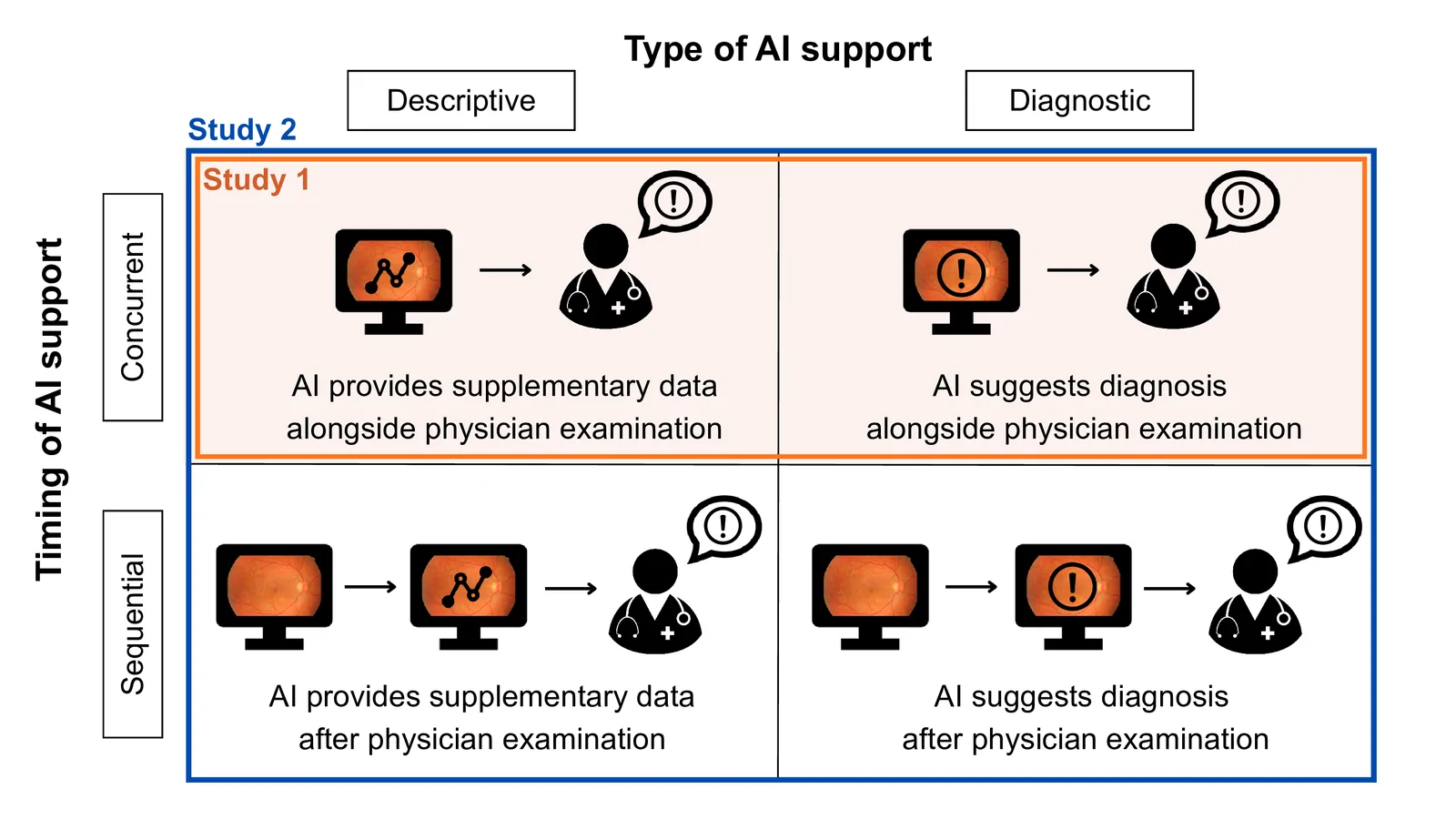
Overview of main experimental conditions in study 1 (orange box) and study 2 (blue box). Two different types of AI support were examined in studies 1 and 2, namely, descriptive AI support and diagnostic AI support. Two different timings of AI support were examined in study 2, namely, concurrent and sequential timing. Both studies additionally investigated a baseline condition (no AI support), which is not shown in this figure.

The 2 main studies were informed by a priori power analyses and included large samples (study 1: N=489; study 2: N=570). Study 1 focused on comparing different types of AI support. Study 2 was conducted to replicate study 1 and additionally examine the effect of AI support timing (Figure 2). Quantitative outcome measures were complemented by qualitative analyses of open-ended responses to provide deeper insight into participants’ reasoning and trust-related considerations.

Both studies were preregistered and conducted in German using Qualtrics (Qualtrics LLC), a web-based survey platform. Preregistrations, data, and analysis scripts are available on Open Science Framework [61]. Reporting follows the GROVE (Guideline for Reporting of Vignette Experiments) guidelines for online vignette experiments in health care contexts [56], with the completed checklist provided in Checklist 1.

### Study 1: Type of AI Support

#### Study 1 Overview

Study 1 examined how the type of physician AI support used in hybrid medical decision-making influences potential patients’ trust, perceptions, and behavioral intentions. To this end, we used a between-subjects design, in which participants were randomly assigned to one of three experimental conditions: (1) physician without AI support (*No AI*), (2) physician with descriptive AI support, which provides only visualizations or additional analyses (*Descriptive AI*), or (3) physician with diagnostic AI support, where the AI system also suggests a preliminary diagnosis to the physician (*Diagnostic AI*).

We expected that trust in the medical decisions (hypothesis [H]1a), perceived provider trustworthiness (H1b), perceived uniqueness neglect (H1c), and willingness to follow the advice (H1d) would not be rated differently in the conditions *No AI* and *Descriptive AI* but less favorably in the condition *Diagnostic AI*.

#### Participants

Participants were recruited via a subject pool comprising employees who were enrolled as part-time students at a German university alongside their regular employment. Data collection took place between March and April 2025 (median participation time: 26 minutes). Prior to data collection, we conducted a power analysis using data simulations in R (R Core Team) with the package simr [62], based on small to medium effects observed in the pilot study (approximately Cohen *d*=0.10‐0.52). The simulations indicated that a total sample size of 450 would provide adequate power (approximately 0.80 across outcomes) at a significance level of α=.05.

#### Design and Procedure

**Figure 3.**
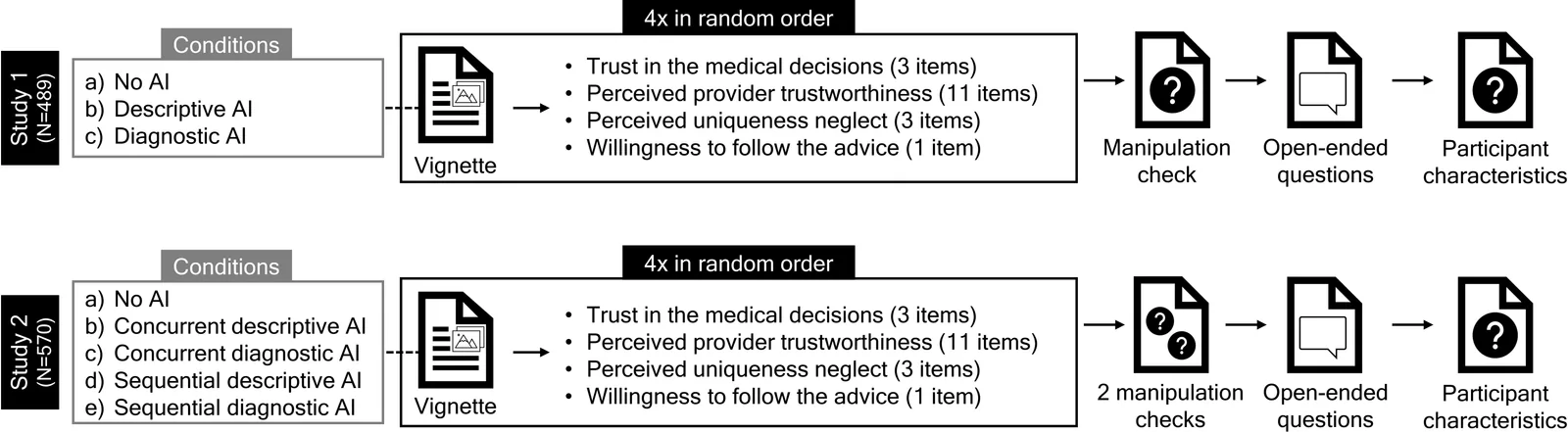
Overview of the design and procedure of study 1 and study 2. Both studies followed a similar procedure, where participants read 4 vignettes in randomized order and responded to the outcome variables after each vignette. At the end of the experiment, participants completed 1 (study 1) or 2 (study 2) manipulation checks, answered open-ended questions, and provided sociodemographic details. Study 1 examined 3 experimental conditions, while study 2 examined 5 experimental conditions.

Each participant read 4 vignettes in randomized order, each describing a different medical consultation (Figure 3). The consultations described in the vignettes varied in symptoms, clinical settings, diagnoses, and medical recommendations, as detailed in the subsequent section. Presenting multiple scenarios allowed us to assess participants’ perceptions across a broader range of realistic medical contexts and AI use cases. This approach increases reliability by reducing the influence of idiosyncratic features of any single vignette.

The experimental condition remained consistent across all vignettes for each participant to make the core manipulation less transparent. This design avoided direct comparisons between the experimental conditions and reduced the risk that participants artificially align their responses. Participants were asked to imagine themselves as patients in the described situation and then responded to the outcome variables. After reading all vignettes and answering the outcome measures for each, participants completed a manipulation check in which they indicated whether the physician (1) did not use an AI system, (2) used an AI system for visual support only, or (3) used an AI system also for diagnostic support. Manipulation checks were administered without allowing participants to revisit the vignette text. To supplement the quantitative data, participants then answered open-ended questions to provide deeper insight into their thoughts and feelings about hybrid medical decision-making. Finally, participant characteristics were collected.

#### Vignettes

Our vignettes described ophthalmology consultations—a field in which AI-CDSS hold great potential and offer diverse use cases [44]. The vignettes were codeveloped with ophthalmologists to ensure clinical plausibility. Together, we identified areas in which AI tools are already being used or hold potential for future application. We selected the use cases to reflect different clinical stakes and settings. The 4 selected use cases were screening (diabetic retinopathy), emergency (retinal detachment), risk prediction (oculomics), and follow-up (glaucoma). Two ophthalmologists reviewed and refined the written vignettes, with particular attention to the realism of the diagnostic procedures and clinical interactions described.

The vignettes were written in the first-person perspective to facilitate participant immersion and because prior research has shown that perceptions of AI in medical decision-making differ when individuals evaluate care for themselves compared with care for others [27,33]. Each vignette detailed the reason for the visit, diagnostic procedures (including retinal imaging), and how the physician derived the medical decisions, varying by experimental condition. In the condition *No AI*, the physician analyzed the retinal images and determined the diagnosis and medical advice without AI support. In the condition *Descriptive AI*, the AI support system highlighted features in the retinal images to support physician interpretation. The physician then determined the diagnosis and medical advice. In the condition *Diagnostic AI*, the AI support system also suggested a preliminary diagnosis, which the physician reviewed. The physician then determined the final diagnosis and medical advice.

**Figure 4.**
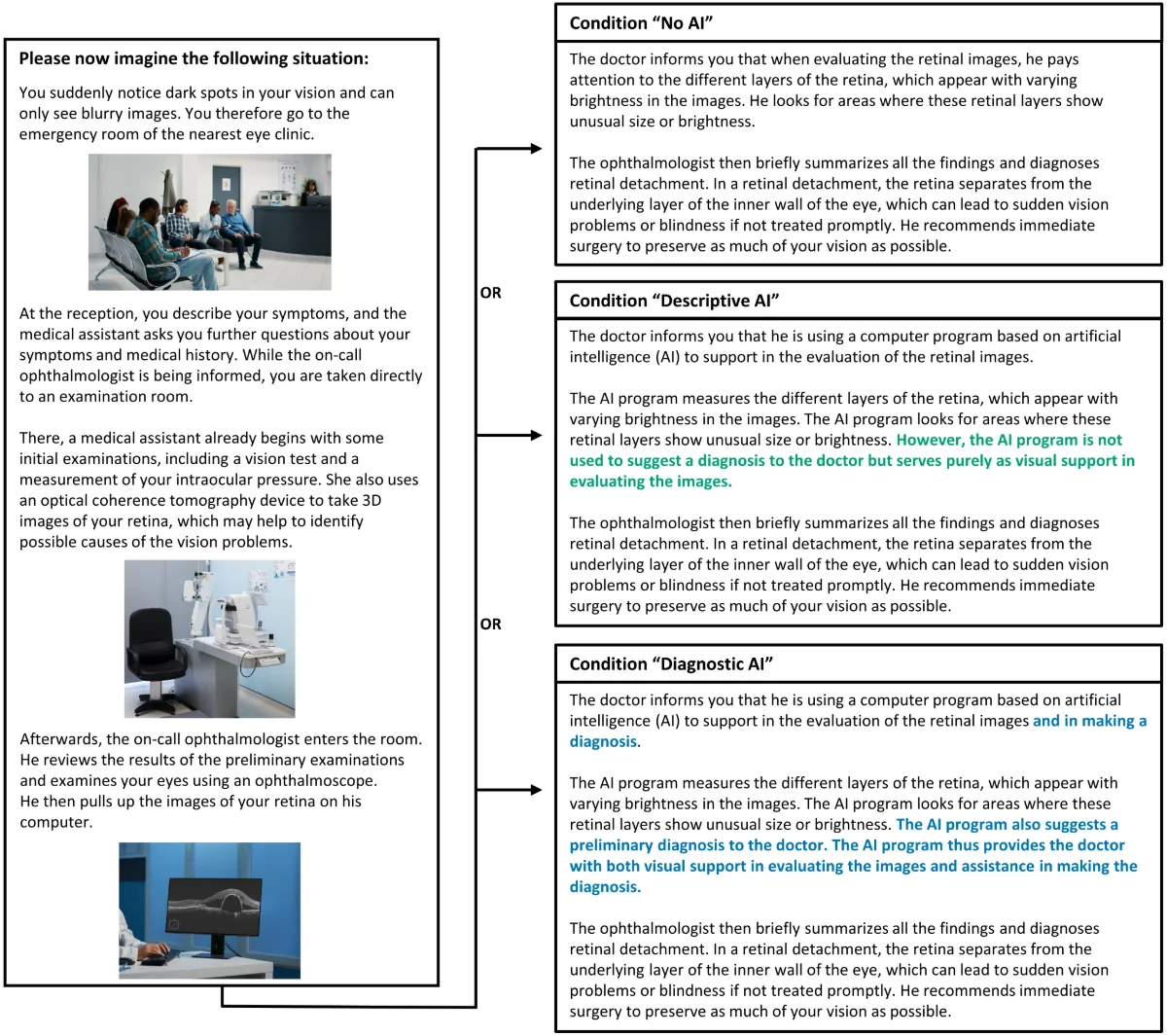
Example vignette from study 1. Whereas each vignette’s introductory text and images were the same across all experimental conditions, the vignettes continued differently depending on the experimental condition. To highlight the differences between the 2 hybrid conditions, text unique to the condition *Descriptive AI* is shown in green and text unique to the condition *Diagnostic AI* is shown in blue. In the actual studies, participants viewed the vignettes without such highlights.

Note that the manipulations were implemented in a relatively subtle manner to reduce demand characteristics and avoid participants deducing the overall study goal. For example, no condition labels or bolded text were used. As it was not the aim of this study to examine the effect of matching versus contradicting assessments by the physician and AI, the vignettes in the hybrid conditions did not contain information about the specific results of the AI analyses nor whether they confirmed or contradicted the physician’s assessment. Furthermore, the consultations were set in unfamiliar medical settings (eg, a new clinic or regular physician on leave) to avoid that participants’ associations with their own, real doctors influenced the results. Perceived vignette realism was captured by 4 items (eg, “The situation was believable.” “I was able to imagine the situation.”) on 7-point Likert scales, with higher values indicating higher realism. Mean ratings, ranging between 5.50 (risk prediction) and 5.62 (emergency), indicated that participants were able to imagine the situations sufficiently well (Table S1 in Multimedia Appendix 2). Figure 4 displays an example vignette including the different experimental versions. Multimedia Appendix 3 contains all vignettes.

#### Measures

The following four outcome variables were assessed, capturing key components of trust [63] and aligning with outcome measures used in prior research on AI-supported medical decision-making [22,29,33,40]:

1. *Trust in the medical decisions* was measured with 3 self-constructed items (eg, “I trust the medical decisions made in this situation.”). Cronbach α values ranged between α=.92 and α=.95 across vignettes, indicating high internal consistency.
2. *Perceived provider trustworthiness* was measured with 11 items adapted from McKnight et al [64] (eg, “Overall, this medical provider is a capable and competent provider of medical services.”). Internal consistency was high for every vignette (α=.97).
3. *Perceived uniqueness neglect* was measured with 3 items adapted from Yokoi et al [29] (eg, “This medical provider would not take my unique circumstances into account.”). Cronbach α values ranged between α=.83 and α=.85 across vignettes.
4. *Willingness to follow the advice* was measured with 1 item, following Reis et al [22] (“How likely is it that you would follow the medical advice?”).

All items were rated on 7-point Likert scales, with higher scores reflecting higher levels of the measured constructs. English scales were back-translated into German. Multimedia Appendix 4 provides an overview of all items. Participants also answered open-ended questions addressing influencing factors on their quantitative responses (Q1), their appraisal of the physician’s use of AI in the situations (Q2), and suggestions for increasing trust (Q3).

#### Analyses

We fitted linear mixed-effects models (LMMs) in R (version 4.4.0) using the lme4 package [65], with *Condition* as a fixed effect and *Participant* as a random intercept to account for the nested data structure. Significant effects were followed by pairwise comparisons of estimated marginal means (EMMs) using Bonferroni-Holm correction for multiple testing (emmeans [66]). As robustness checks, we additionally corrected the family-wise error rate across the 4 outcomes using Bonferroni-Holm correction on the omnibus tests, repeated the main analyses in intention-to-treat samples including participants who failed the manipulation checks, and conducted exploratory analyses examining the specific vignette as an additional main effect and its potential interaction with the AI support condition. Note that the vignettes differed on several dimensions at once, including clinical setting, symptom severity, diagnostic procedures, and invasiveness of recommended treatments. Therefore, these exploratory analyses of specific vignette effects should be interpreted as tests of broad scenario–level differences rather than as isolating the specific effects of stakes, acuity, or any other single vignette feature.

Analysis of the open-ended responses followed a 3-step process. First, in line with prior studies that used AI to analyze large qualitative datasets [67,68], we used ChatGPT-4 (OpenAI) to provide an initial list of candidate recurring themes across the 489 responses for each of the 3 open-ended questions. The model was prompted in German using the original response language, and its role was limited to preliminary theme surfacing. To enhance transparency, the prompts and suggested themes are included in Multimedia Appendix 5. The use of a large language model at this stage was motivated by the size of the qualitative dataset. The output provided an initial overview of potential themes but served only as a starting point. Human coders carefully reviewed and refined the themes in the subsequent steps, ensuring that themes and interpretations are grounded in the original data. Second, initial codebooks for Q1-Q3 were developed by grouping themes and removing redundancies. Third, a human rater applied the initial coding scheme, coding which themes were present in each response. During this process, the coding scheme was iteratively refined to capture all relevant content and reapplied. For example, when new themes emerged inductively, these were added to the codebook. To mitigate potential hallucinations or model bias, ChatGPT was not used for final coding, interpretation, or quote selection. All reported themes resulted from human coding. A second human rater independently coded 15% of the responses for each question to assess the reliability of the final coding scheme. Interrater agreement was substantial for all 3 questions, with Cohen κ=0.71 for Q1, κ=0.71 for Q2, and κ=0.71 for Q3 [69].

Additionally, we subjected responses to Q2 to exploratory sentiment analysis using the Hugging Face checkpoint cardiffnlp/twitter-xlm-roberta-base-sentiment, a multilingual XLM-RoBERTa-base model [70], in Python (version 3.11.11; Python Software Foundation). The underlying XLM-T model was trained on approximately 198 million tweets and fine-tuned for 3-class sentiment classification on an 8-language benchmark that includes German. In the source paper [70], German performance reached macro-*F*_1_ values of 76.13 in monolingual evaluation and 77.35 in multilingual evaluation on held-out test data. For each response, the model produced 3 class logits, which were transformed using the softmax function into normalized scores for the negative, neutral, and positive sentiment classes (ranging from 0 to 1). These continuous class scores were then compared across conditions using 1-way ANOVAs, followed by 2-tailed Bonferroni-Holm–adjusted *t* tests when significant effects were observed. Results of the sentiment analysis are provided in Tables S2 and S3 in Multimedia Appendix 2. Because the model was not trained and validated on health care–related text specifically, its validity for the present task is unclear. Therefore, the sentiment analyses were treated as exploratory, and results should be interpreted with caution.

### Study 2: Type and Timing of AI Support

#### Study 2 Overview

Study 2 sought to replicate findings from study 1 and to extend them by varying not just the type but also the timing of AI support. The 2 hybrid conditions in study 1 exemplified a concurrent workflow, in which AI support was reviewed alongside the primary clinical information. Study 2 therefore crossed *AI Support Type* (descriptive vs diagnostic) with *AI Support Timing* (concurrent vs sequential), yielding 4 hybrid configurations in addition to the no-AI baseline (Figure 2).

We hypothesized that evaluations would not be rated differently between *No AI* and *Concurrent Descriptive AI* but lower for *Concurrent Diagnostic AI* across all 4 outcome variables (H2a-H2d). Additionally, we explored whether *AI Support Type* and *AI Support Timing* interactively influence participants’ evaluations of AI-assisted medical decisions.

#### Participants

Participants were recruited via subject pools of 4 German universities, with the majority sampled again from the pool of employees enrolled as part-time students. Data collection for study 2 took place between June and October 2025 (median participation time: 25 minutes). We estimated the required sample size for study 2 based on parameters from study 1. We preregistered a target sample size of 500 to achieve a power of 0.80, given α=.05.

#### Design and Procedure, Vignettes, and Measures

Participants were randomly assigned to 1 of 5 between-subjects conditions: *No AI*, *Concurrent Descriptive AI*, *Concurrent Diagnostic AI*, *Sequential Descriptive AI*, or *Sequential Diagnostic AI* (Figure 3). We updated the vignettes from study 1 to distinguish between concurrent and sequential use of physician AI support. Mean ratings of vignette realism, ranging between 5.52 (screening) and 5.72 (emergency), indicated that participants were able to imagine the situations sufficiently well (Table S1 in Multimedia Appendix 2).

Two manipulation checks were applied. First, participants indicated whether the physician (1) did not use an AI system, (2) used an AI system for visual support only, or (3) used an AI system also for diagnostic support. Second, participants in the hybrid conditions additionally indicated whether the physician communicated their own assessment before reviewing the AI output (*sequential*) or not (*concurrent*). Otherwise, design and procedure mirrored those of study 1. We used the same quantitative measures as in study 1. Internal consistency per vignette was again high for all composite scales (trust in the medical decisions: α=.91-.96, perceived provider trustworthiness: α=.97, and perceived uniqueness neglect: α=.84-.85). Participants were also asked the open-ended questions Q2 and Q3 from study 1.

#### Analyses

Given that the condition *No AI* cannot be categorized by *AI Support Timing*, our design was partially factorial. Therefore, we preregistered 2 complementary analyses using LMMs for each dependent variable. To examine main and interaction effects of *AI Support Type* and *AI Support Timing*, we used a 2x2 factorial model, which included only the 4 hybrid conditions. To compare all 5 conditions, we additionally used an all-conditions model (as in study 1). In both analyses, *Participant* was entered as random intercept. The analyses were followed up with Bonferroni-Holm–adjusted comparisons of EMMs. We conducted the same robustness checks as in study 1.

Open-ended responses to Q2 and Q3 were qualitatively analyzed using a deductive-inductive approach. A coder, who was experienced in qualitative analyses and who had not been involved in the analyses for study 1, applied the coding scheme developed in study 1 to the responses from study 2 while remaining open to modifications if new themes emerged. Additionally, open-ended responses to Q2 were subjected to sentiment analysis following the same procedure as in study 1. These results are reported in Tables S16 and S17 in Multimedia Appendix 2.

### Ethical Considerations

The project was approved by the Shared Ethics Committee for Bavarian Universities of Applied Sciences (GEHBa-202405-V-185). The studies were conducted in accordance with the principles of the Declaration of Helsinki. Participants provided informed consent prior to participation and received 0.5 hours of research participation credit as compensation. The study data were collected anonymously. All vignette images depicting people were licensed stock images; the individuals shown in these images were not study participants, patients, or members of the research team.

## Results

### Study 1: Type of AI Support

#### Sample Demographics

Of 625 respondents, 8 (1%) were excluded because they completed the survey more than 2 times faster than the median duration (as recommended by Leiner [71]), 33 (5%) were excluded because they failed at least 1 of 2 attention checks, and 95 (15%) were excluded because they failed the manipulation check. The final sample for study 1 consisted of 489 participants (n=336 females, 69%) with a mean age of 25.6 (SD 4.6) years, ranging from 18 to 49 years. Detailed demographics are reported in Table 1.

**Table 1.** Sample characteristics for study 1 (N=489) and study 2 (N=570).

Characteristic	Study 1	Study 2
Age (years), mean (SD)	25.6 (4.6)	25.07 (5.3)
Sex, n (%)		
Male	152 (31.1)	158 (27.7)
Female	336 (68.7)	410 (71.9)
Diverse	1 (0.2)	1 (0.2)
Prefer not to say	0 (0.0)	1 (0.2)
Country, n (%)		
Germany	487 (99.6)	566 (99.3)
Austria	1 (0.2)	2 (0.4)
Other	1 (0.2)	2 (0.4)
Education, n (%)		
Middle school degree or equivalent	10 (2)	10 (1.8)
High school degree	223 (45.6)	305 (53.5)
Vocational training	241 (49.3)	212 (37.2)
University degree	14 (2.9)	41 (7.2)
PhD	0 (0.0)	0 (0.0)
Other	1 (0.2)	2 (0.4)
Employment, n (%)		
Full-time employed	235 (48.1)	219 (38.4)
Part-time employed	112 (22.9)	115 (20.2)
Student	134 (27.4)	227 (39.8)
Unemployed or looking for work	0 (0.0)	2 (0.4)
Retired	0 (0.0)	0 (0.0)
Parental leave	6 (1.2)	3 (0.5)
Sabbatical	0 (0.0)	2 (0.4)
Other	2 (0.4)	2 (0.4)

#### Comparisons Between Conditions

EMMs and results from the model comparisons (likelihood ratio tests) are reported in Table 2. Including *Condition* as a fixed effect significantly improved model fit compared with a null model for all outcomes except willingness to follow the advice, indicating that the type of AI involvement affected all outcomes but this one. When controlling for family-wise error across the omnibus tests using Bonferroni-Holm correction, the pattern of results remained unchanged (Table S4 in Multimedia Appendix 2). Results from all (including nonsignificant) pairwise comparisons can be found in Tables S5-S8 in Multimedia Appendix 2.

**Table 2.** Condition effects and EMMs^a^ for each of the 4 outcome variables in study 1 (N=489), using linear mixed-effects models with *Condition* as a fixed effect and *Participant* as a random intercept.

Condition		EMMs (95% CI)	SE	Likelihood ratio test^b^
Trust in the medical decisions				*χ*²_2_=13.25; *P<*.001
No AI		5.28 (5.14-5.43)	0.07	
Descriptive AI		5.37 (5.23-5.52)	0.07	
Diagnostic AI		5.00 (4.85-5.15)	0.08	
Perceived provider trustworthiness				*χ*²_2_=22.71; *P<*.001
No AI		5.33 (5.19-5.48)	0.07	
Descriptive AI		5.40 (5.25-5.55)	0.07	
Diagnostic AI		4.93 (4.78-5.08)	0.08	
Perceived uniqueness neglect				*χ*²_2_=8.96; *P=*.01
No AI		2.96 (2.80-3.12)	0.08	
Descriptive AI		2.93 (2.76-3.09)	0.08	
Diagnostic AI		3.24 (3.08-3.40)	0.08	
Willingness to follow the advice				*χ*²_2_=3.11; *P=*.21
No AI		5.55 (5.40-5.70)	0.08	
Descriptive AI		5.62 (5.47-5.78)	0.08	
Diagnostic AI		5.42 (5.27-5.58)	0.08	

a EMMs: estimated marginal means.

b Comparison of a model with *Condition* as fixed effects to a null model without this term.

In the condition *Diagnostic AI*, trust in the medical decisions was rated significantly lower than in the conditions *Descriptive AI* (*t*_486_=3.51; *P*=.002) and *No AI* (*t*_486_=2.67; *P*=.02). A similar pattern was observed for perceived provider trustworthiness, with *Diagnostic AI* rated significantly lower than *Descriptive AI* (*t*_486_=4.46; *P*<.001) and *No AI* (*t*_486_=3.81; *P*<.001). For perceived uniqueness neglect, where higher scores indicate greater concerns about lack of individualized consideration, *Diagnostic AI* received significantly higher ratings than

*Descriptive AI* (*t*_486_=2.72; *P*=.02) and *No AI* (*t*_486_=2.47; *P*=.03). *Descriptive AI* and *No AI*, however, did not differ significantly from each other on any of these outcomes (all *P*≥.39). No significant differences emerged between any of the conditions regarding willingness to follow the advice (all *P*≥.25). These results therefore support H1a-H1c but not H1d.

Intention-to-treat analyses including participants who failed the manipulation checks showed a largely similar overall pattern. However, effects for perceived uniqueness neglect failed to reach significance, suggesting that this effect depends more strongly on successful processing of the manipulation and should therefore be interpreted more cautiously. Full results are reported in Tables S9-S13 in Multimedia Appendix 2.

In exploratory analyses, adding *Vignette* as a main effect significantly improved model fit for all outcomes, and these effects remained significant after correction for family-wise error across outcomes. The vignette *follow-up* (*glaucoma*) generally elicited less favorable evaluations, whereas the vignettes *screening* (*diabetic retinopathy*) and *emergency* (*retinal detachment*) were evaluated somewhat more favorably across outcomes. By contrast, adding the interaction term *Condition* × *Vignette* did not significantly improve model fit for any outcomes, suggesting that the experimental effects were stable across scenarios (Table S14 in Multimedia Appendix 2).

#### Qualitative Results

Table S15 in Multimedia Appendix 2 summarizes recurring themes for influencing factors (Q1), appraisal of AI support (Q2), and approaches to increasing trust (Q3). Given the aim of this work, we focus particularly on AI-related aspects among these. The open-ended responses suggest that several aspects shape potential patients’ trust in hybrid medical decision-making. These can be roughly categorized as relating to the medical situation, the physician, the participants themselves, and the AI system and dynamics of the physician-AI interaction.

Overall, several participants acknowledged the potential of AI in medical decision-making, for example, to help prevent human oversights. However, many participants were concerned that physicians would overrely on AI recommendations.

*I can imagine that when one is emotionally stressed or irritable, as a doctor one might only tell the patient the AI recommendation, even if it is possibly incorrect, because one wants to get through the work or so*.

*It’s difficult. On the one hand, it’s good if it really only serves as support; however, people rely on AI very quickly, which in turn could lead in the wrong direction*.

As physicians were considered more capable of holistically assessing patients’ unique cases than AI, participants preferred AI-CDSS that play only a smaller role in the diagnostic process over AI-CDSS that provide diagnostic support—consistent with the quantitative results.

*As support, AI can be useful in such situations; however, I believe that the uniqueness of diseases cannot be determined by an AI. A doctor should always make a diagnosis on their own*.

*Perhaps the AI could have only sorted the progression/status or images, but left the diagnosis and evaluation to the doctor. That would make me feel more secure*.

Ultimately, the physician’s interaction with the AI system played an important role. For example, participants considered the physicians’ assumed AI competence and whether they appeared to critically examine the AI output.

*Assuming that the AI system has been adequately tested before use, I am less concerned about the technology itself and more about how it is applied. It would be important to me that the technology is used only as an aid, that the information is thoroughly reviewed afterwards*.

*For me it’s essential that the physician questions the results and does not blindly trust the AI*.

Moreover, several participants expressed a need for more information about the AI system itself, its results, and its usage.

*[Trust would be higher] if the doctor had explained the AI to me in detail, especially focusing on the benefits*.

Another recurring theme was the wish for the use of AI-CDSS as an independent “second opinion” for the physician. Participants described a preferred decision-making workflow where physicians first review all diagnostic information and only look at the AI output after having formed their own assessment, so that potentially incorrect AI output does not bias their clinical judgment prematurely.

*That the doctor states or records their diagnosis/recommendation first and then is shown the AI’s diagnosis/recommendation*.

*Express their own diagnosis or assumption first and not be primed by the AI’s suggestion*.

Overall, the qualitative data suggest that many participants were open to physicians using AI support but only under certain conditions. For example, they valued AI only as an augmentation of the physician’s expertise and voiced concerns about overreliance on AI results. Participants expressed that transparency and the way physicians use AI-CDSS are crucial. The latter includes both the type of AI support (ie, for which tasks) and when that support is used in the clinical decision-making workflow. From their perspective, careful choices about the support type and timing can help safeguard against AI output that is incorrect or overlooks patients’ individual circumstances.

### Study 2: Type and Timing of AI Support

In study 1, diagnostic AI support reduced trust-related evaluations relative to descriptive support, and participants spontaneously indicated a preference for workflows in which the physician forms an assessment before receiving AI support. Study 2 examines whether this qualitative finding also appears in quantitative measures.

#### Sample Demographics

Of 850 respondents, 53 (6%) were excluded because they completed the survey more than 2 times faster than the median duration, 38 (4%) were excluded because they failed at least 1 attention check, 81 (10%) were excluded because they failed the first manipulation check, and 108 (13%) were excluded because they failed the second manipulation check. The final sample consisted of 570 participants (n=410 females, 72%) with a mean age of 25.1 (SD 5.3) years, ranging from 18 to 58 years. Detailed demographics are reported in Table 1.

#### Main and Interaction Effects of AI Support Type and Timing

**Figure 5.**
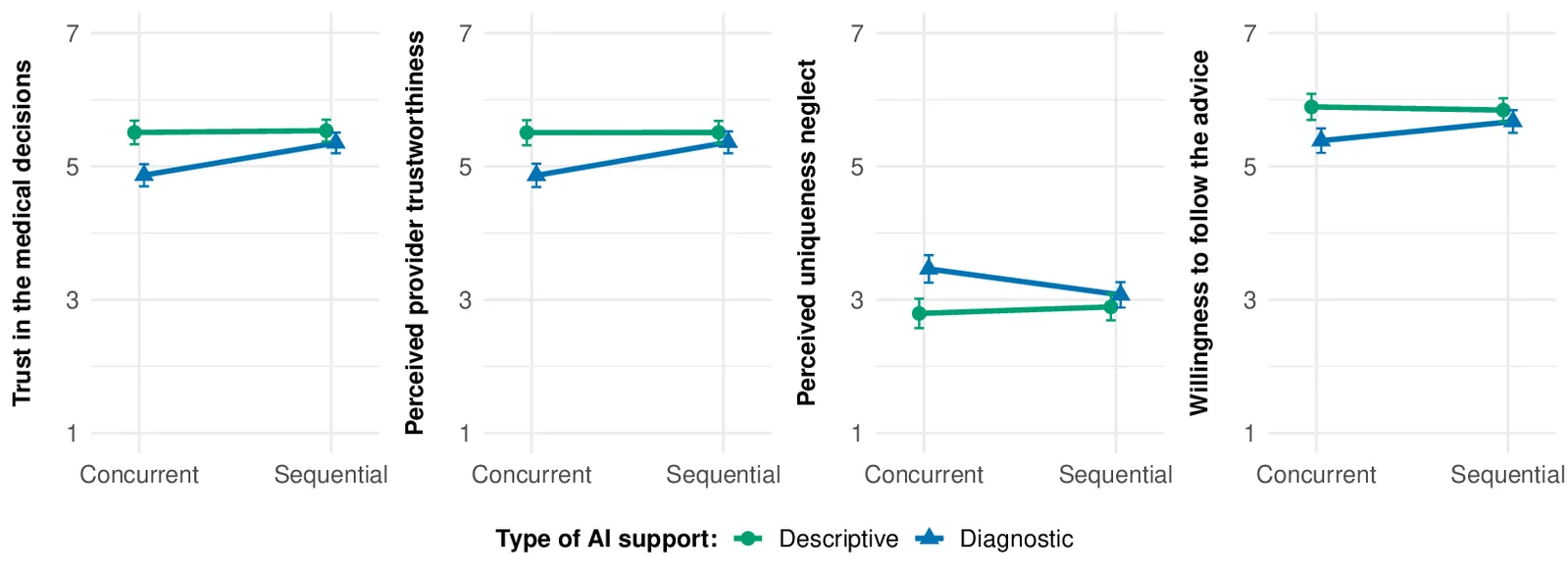
Interaction effects for type of AI support and timing of AI support. Error bars are 95% CIs around estimated marginal means. Only data from hybrid conditions were used in these analyses (N=440). The interaction effect was significant for trust in the medical decisions (*t*_436_=2.71; *P=*.007), perceived provider trustworthiness (*t*_436_=2.78; *P=*.006), and perceived uniqueness neglect (*t*_436_=−2.34; *P=*.02) but not for willingness to follow the advice (*t*_436_=1.81; *P=*.07).

For trust in the medical decisions, perceived provider trustworthiness, and perceived uniqueness neglect, 2x2 LMM analyses found a significant main effect of *AI Support Type*, which was qualified by a significant interaction effect (Table 3). For willingness to follow the advice, a significant main effect of *AI Support Type* emerged, but the interaction effect failed to reach statistical significance (*P=*.07). The interaction plots are shown in Figure 5. When controlling for family-wise error across the omnibus tests using Bonferroni-Holm correction, the pattern of results remained unchanged (Table S4 in Multimedia Appendix 2).

Intention-to-treat analyses including participants who failed the manipulation checks produced the same pattern of results (Table S18 in Multimedia Appendix 2). In exploratory analyses investigating main and interaction effects of vignette, adding *Vignette* as a main effect significantly improved model fit for all outcomes, also after correcting for family-wise error. Generally, the vignette *follow-up* (*glaucoma*) elicited less favorable evaluations, whereas *screening* (*diabetic retinopathy*) and *emergency* (*retinal detachment*) tended to be evaluated more favorably. By contrast, adding the higher-order interaction terms involving *Vignette* did not improve model fit for any outcome, suggesting that the effects of AI support type and timing were stable across scenarios (Table S19 in Multimedia Appendix 2).

**Table 3.** Main and interaction effects for each of the 4 outcome variables in study 2, using 2x2 linear mixed-effects models with *AI Support Type* and *AI Support Timing* as fixed effects and *Participant* as random intercept. Only data from hybrid conditions (N=440) were used in these analyses.

Condition		Estimate	SE	*t* test (*df*)	*P* value
Trust in the medical decisions					
Intercept		5.51	0.09	60.73 (436)	.<.001
AI Support Type^a^		−0.64	0.12	−5.17 (436)	<.001
AI Support Timing^b^		0.03	0.12	0.22 (436)	.82
AI Support Type x AI Support Timing		0.46	0.17	2.71 (436)	.007
Perceived provider trustworthiness					
Intercept		5.51	0.10	57.45 (436)	<.001
AI Support Type^a^		−0.64	0.13	−4.90 (436)	<.001
AI Support Timing^b^		<.01	0.13	0.02 (436)	.98
AI Support Type x AI Support Timing		0.49	0.18	2.78 (436)	.006
Perceived uniqueness neglect					
Intercept		2.79	0.11	24.96 (436)	<.001
AI Support Type^a^		0.67	0.15	4.36 (436)	<.001
AI Support Timing^b^		0.10	0.15	0.65 (436)	.52
AI Support Type x AI Support Timing		−0.49	0.21	−2.34 (436)	.02
Willingness to follow the advice					
Intercept		5.89	0.10	59.14 (436)	<.001
AI Support Type^a^		−0.51	0.14	−3.72 (436)	<.001
AI Support Timing^b^		−0.05	0.13	−0.36 (436)	.72
AI Support Type x AI Support Timing		0.34	0.19	1.81 (436)	.07

a *Descriptive AI* served as reference category.

b *Concurrent Timing* served as reference category.

#### Comparisons Between Conditions

EMMs and results of the likelihood ratio tests are displayed in Table 4. Results from LMMs including all 5 conditions showed that including *Condition* as a fixed effect significantly improved model fit compared with a null model for all outcomes (all *P* values <.001). When controlling for family-wise error across the omnibus tests using Bonferroni-Holm correction, the pattern of results remained unchanged (Table S4 in Multimedia Appendix 2). Results from all (including nonsignificant) pairwise comparisons can be found in Tables S20-S23 in Multimedia Appendix 2.

Regarding trust in the medical decisions, *Concurrent Diagnostic AI* received significantly lower ratings than *Concurrent Descriptive AI* (*t*_565_=5.09; *P*<.001), *Sequential Descriptive AI* (*t*_565_=5.57; *P*<.001), *Sequential Diagnostic AI* (*t*_565_=4.13; *P*<.001), and *No AI* (*t*_565_=2.65; *P*=.04). Compared with the baseline condition *No AI*, both *Concurrent Descriptive AI* (*t*_565_=2.76; *P*=.04) and *Sequential Descriptive AI* (*t*_565_=3.14; *P*=.01) received significantly higher ratings of trust in the medical decision. Together, these results partially support H2a.

Also regarding perceived trustworthiness of the medical provider, *Concurrent Diagnostic AI* evoked less favorable ratings than all other conditions. Specifically, *Concurrent Diagnostic AI* was rated significantly lower than *Concurrent Descriptive AI* (*t_565_*=4.95; *P*<.001), *Sequential Descriptive AI* (*t_565_*=5.22; *P*<.001), *Sequential Diagnostic AI* (*t*_565_=4.12; *P*<.001), and *No AI* (*t_565_*=3.52; *P*=.003). These results support H2b.

The reverse pattern emerged for perceived uniqueness neglect. Here, *Concurrent Diagnostic AI* was rated significantly higher than *Concurrent Descriptive AI* (*t*_565_=4.46; *P*<.001), *Sequential Descriptive AI* (*t*_565_=3.99; *P*<.001), *Sequential Diagnostic AI* (*t*_565_=2.79; *P*=.04), and *No AI* (*t*_565_=3.84; *P*=.001). These results support H2c.

Regarding willingness to follow the medical advice, *Concurrent Diagnostic AI* also received significantly lower ratings than *Concurrent Descriptive AI* (*t*_565_=3.74; *P*=.002) and *Sequential Descriptive AI* (*t*_565_=3.55; *P*=.004), whereas no other contrasts for this outcome variable reached statistical significance. These results partially support H2d.

Overall, the results mirror those of the 2x2 LMMs: When the timing of AI use in the decision-making workflow was framed as concurrent, *Diagnostic AI* received significantly less favorable evaluations than *Descriptive AI* on all outcomes, replicating results from study 1. When the timing of AI use in the decision-making workflow was framed as sequential, the 2 types of AI support did not differ significantly from each other anymore. This was driven by improved ratings of diagnostic AI support in sequential decision-making compared with concurrent decision-making.

Intention-to-treat analyses including participants who failed the manipulation checks yielded a largely unchanged pattern of results, with the differences between the conditions *Diagnostic AI* and *Descriptive AI* remaining significant in concurrent workflows and not significant in sequential workflows for all outcomes (Tables S24-S28 in Multimedia Appendix 2). Exploratory analyses investigating main and interaction effects of *Vignette* showed significant main effects for all outcomes. Again, the vignette *follow-up* (*glaucoma*) tended to elicit lower trust-related evaluations. However, no interaction effects were significant after correction for family-wise error. This suggests that participants responded differently to the clinical scenarios overall, whereas the relative pattern of condition effects was stable across vignettes (Table S29 in Multimedia Appendix 2).

**Table 4.** Condition effects and EMMs^a^ for each of the 4 outcome variables in study 2, using linear mixed-effects models with *Condition* as a fixed effect and *Participant* as a random intercept. Data from all conditions (N=570) were used in these analyses.

Condition	EMM (95% CI)	SE	Likelihood ratio test^b^
Trust in the medical decisions			*χ*²_4_=40.08; *P<*.001
No AI	5.17 (5.02-5.33)	0.08	
Concurrent descriptive AI	5.51 (5.33-5.69)	0.09	
Sequential descriptive AI	5.54 (5.37-5.70)	0.08	
Concurrent diagnostic AI	4.87 (4.70-5.04)	0.09	
Sequential diagnostic AI	5.35 (5.19-5.51)	0.08	
Perceived provider trustworthiness			*χ*²_4_=34.70; *P<*.001
No AI	5.28 (5.13-5.44)	0.08	
Concurrent descriptive AI	5.51 (5.32-5.69)	0.09	
Sequential descriptive AI	5.51 (5.34-5.68)	0.09	
Concurrent diagnostic AI	4.86 (4.69-5.04)	0.09	
Sequential diagnostic AI	5.36 (5.20-5.52)	0.08	
Perceived uniqueness neglect			*χ*²_4_=25.42; *P<*.001
No AI	2.93 (2.75-3.11)	0.09	
Concurrent descriptive AI	2.79 (2.58-3.01)	0.11	
Sequential descriptive AI	2.89 (2.70-3.09)	0.10	
Concurrent diagnostic AI	3.46 (3.26-3.66)	0.10	
Sequential diagnostic AI	3.07 (2.89-3.26)	0.09	
Willingness to follow the advice			*χ*²_4_=20.15; *P<*.001
No AI	5.54 (5.38-5.71)	0.08	
Concurrent descriptive AI	5.89 (5.70-6.09)	0.10	
Sequential descriptive AI	5.84 (5.67-6.02)	0.09	
Concurrent diagnostic AI	5.38 (5.20-5.57)	0.09	
Sequential diagnostic AI	5.67 (5.50-5.84)	0.09	

a EMMs: estimated marginal means.

b Comparison of a model with AI support type and AI support timing as fixed effects to a null model without this term.

#### Qualitative Results

The qualitative findings derived from Q2 and Q3 in study 2 largely mirrored those of study 1, with broadly similar proportions of mentions across themes (Table S30 in Multimedia Appendix 2). No additional themes emerged. Participants again expressed ambivalent views toward AI-supported medical decision-making, recognizing both the potential of AI to support physicians as well as concerns about physician overreliance and the possibility that incorrect AI output could bias the final decision.

*I think it is good, provided that the doctors do not rely too much on the program and perhaps stop looking carefully enough. I would like to have the program as a safety net in case the doctor overlooks something or if something is difficult to recognize with the eye*.

Several responses further suggested that comfort with physician-facing AI support depends on how it is used. For example, one participant in the *Concurrent Descriptive AI* condition noted positively that “It is also very good that no diagnosis is suggested and that the doctor is not actively influenced in their diagnosis.” More specifically, several responses reflected the central quantitative finding of study 2, namely, that sequential workflows were experienced as more reassuring than concurrent ones.

*[I find the use of AI] okay as support, but only after a thorough independent formation of the diagnosis*.

More specifically, participants seemed to value not only the sequential process itself but also the communication of the physician’s independent assessment before consulting AI.

*I do not find the use [of AI] unsettling. On the contrary, in the described situations, but only because the doctors stated their diagnosis beforehand, the AI finding had a purely supportive and positive impact*.

Overall, these qualitative results reinforce the interpretation that evaluations of hybrid medical decisions depend not only on whether AI is used but also on perceptions of the physician-AI collaboration.

## Discussion

### Principal Findings

As AI support systems become more integrated into medical decision-making, it is important to understand how their use is perceived by potential patients. In this paper, we investigated how the type and timing of clinician-facing AI support shape potential patients’ evaluations of the resulting medical decision. Three principal findings emerged.

#### Preference for Descriptive AI Support Over Diagnostic AI Support

Our results suggest that when the physician uses AI support during (concurrent) medical decision-making, patients trust the resulting decision less if the AI system had already suggested a preliminary diagnosis to the physician than if the AI system had provided only supplementary analyses or visualizations. The same applies to the extent to which patients perceive the medical provider to be trustworthy and considerate of their unique circumstances.

Qualitative insights from participants’ open-ended responses complement these quantitative results. For example, participants expressed that deriving a diagnosis requires a holistic consideration of the patients’ case, which encompasses more than the interpretation of medical imaging. Therefore, they preferred AI rather for smaller, supportive tasks but wanted the diagnosis to remain with human expertise. Additionally, participants expressed concerns that incorrect diagnoses suggested by AI could bias physicians’ judgments and thereby compromise the final clinical decision. Together, these qualitative insights suggest that diagnostic AI support is disadvantaged by a “double curse.” First, it is perceived as more likely to produce inadequate recommendations than descriptive AI support, with its diagnostic quality expected to fall short compared with a physician. Second, when such shortcomings occur, diagnostic AI support is seen as more harmful to the final decision than descriptive AI support, because its suggestions may influence physicians’ judgments more directly.

#### Sequential Decision-Making Mitigates Aversion to Diagnostic AI Support

Study 2 revealed that aversion toward diagnostic AI support was eliminated when physicians reviewed AI output only after forming their own independent clinical assessment (ie, in sequential workflows). Participants’ open-ended responses suggest that they view the timing of AI support as a way to reduce the risk of overreliance on AI advice and safeguard the final decision from potentially incorrect AI output, namely, through sequential decision-making.

#### Willingness to Follow the Advice Is Somewhat Less Affected by AI Support Use

In contrast to reported trust and perceptions, participants’ willingness to follow the medical advice was comparably less affected by the use of AI support, remaining stable across conditions in study 1 and showing only modest variation in study 2. While the use of diagnostic AI in a concurrent workflow still lowered participants’ willingness to follow the medical advice compared with some other types of AI involvement in study 2, behavioral intentions seem to be less sensitive to the use of AI support than subjective perceptions and trust. A possible explanation might be that when deciding whether to follow medical advice, other factors are more relevant to patients than how the medical decision was reached.

For example, in our vignettes, the diagnostic procedure always yielded a clinically concerning health result, which required medical action. Consequently, patients may be inclined to follow recommendations for active intervention “to be on the safe side,” even when they feel uneasy about how the decision was reached. This could partly explain the rather high willingness to follow the medical advice across conditions. Such a tendency has also been observed in previous studies [30,72,73]. For example, Detjen et al [30] found that although AI interpretations of mammograms were trusted less than those of physicians, participants were still willing to follow AI recommendations for a biopsy—presumably due to risk aversion. In our study, condition effects might have been more pronounced if the vignettes had described scenarios with favorable health results recommending no immediate action, as suggested by the findings of previous studies [72,74].

At the same time, the possibility of ceiling effects limiting sensitivity to detect differences between conditions should be noted. Our vignette framing may have contributed to strong baseline intentions to follow the medical advice and therefore restricted upper-end variability on this variable. Although we still observed some variance on this variable (SD_Study 1_=1.50, SD_Study 2_=1.49), responses in both studies were skewed toward the upper end of the scale. Accordingly, the comparatively weak condition effects for willingness to follow the medical advice should be interpreted cautiously.

### Comparison With Prior Work

Previous vignette experiments on potential patients’ perception of hybrid medical decisions produced inconsistent results [22,40,42,43]. The present findings provide more nuance by suggesting that trust in hybrid medical decisions depends less on *whether* AI is involved in the decision-making process and more on *how* it is involved (eg, regarding the type and timing of AI use). The observation that the use of AI for *descriptive* support (eg, additional visualizations and information) does not lead to an “AI penalty” regarding trust is in line with those studies that found no difference in evaluations between physician-only and hybrid medical decisions [33,40]. In contrast, the observation that the use of *diagnostic* AI support (in concurrent decision-making workflows) negatively impacts trust and perceptions aligns with those studies that found less favorable evaluations of hybrid decisions than physician-only decisions [22,42,43]. This pattern also aligns with research outside health care suggesting that acceptance of AI is lower for complex, high-stakes tasks that are deemed to require human judgment than for low-stakes tasks requiring rather mechanical skills [50,51,75,76].

Sequential decision-making workflows similarly mitigated “AI penalty” effects, likely due to the assumption that such workflows reduce the risk of overreliance. This expectation aligns with human-AI interaction research showing that requiring users to make an initial assessment before receiving AI advice can reduce overreliance on (incorrect) AI advice [8,53,77]. Recent work in the medical domain also found an evaluative advantage of sequential workflows: In a fictitious malpractice judgment study, laypeople penalized a physician for disagreeing with correct AI advice more when the physician interpreted a computed tomographic scan once after seeing AI advice than when the physician interpreted the scan first without AI advice and then with AI advice [54]. Other studies demonstrated patients’ appreciation of AI for providing a second, independent opinion [18,30]. The same motivation likely underlies the preference for a sequential decision-making workflow in hybrid decision-making: The qualitative results suggested that participants viewed 2 independent assessments to be more reliable than 1 combined. In contrast, concurrent workflows may, from the participants’ perspective, not only undermine the advantage of receiving a second opinion but also introduce the risk that one party unduly biases the other.

A related line of research has demonstrated that the specific AI results—and whether they confirm or contradict the physician’s opinion—influence potential patients’ trust [30,72-74]. The present work complements that line of research by shifting attention from the content of AI output to modifiable workflow features that may serve as levers for improving patient comfort with hybrid decision-making, even when the diagnostic result itself cannot be changed.

At the same time, the effects of such workflow features likely depend in part on how they are communicated to patients. Some of the open-ended responses echoed previous research highlighting the role of transparency and communication in AI-assisted care. For example, several participants expressed the wish for more information, for example, about the AI system itself, its results, or how its output informed the final decision. Similar informational needs have been reported in previous interview studies with patients [18,78,79]. The qualitative findings further highlighted the role of communication as a means to increase transparency about the extent to which both physician and AI assessments align. In addition, several recurring concerns—such as the physician’s competence in using AI appropriately and the possibility of overreliance on AI—suggest that communication may be an important means of strengthening trust, in line with earlier findings [17,78].

### Practical Implications

At present, there is no established or regulated standard for clinical workflows involving AI-CDSS or for how their use should be communicated to patients [78,80-83]. Against this background, the present findings have several implications for the use of AI-CDSS during medical consultations. Sequential decision-making workflows may help alleviate patient concerns about diagnostic AI support by ensuring that the physician’s first—and ideally final—clinical judgment remains independent of potential AI-suggested misdiagnoses. However, such workflows may reduce efficiency, which is a major motivation for adopting AI-CDSS in the first place [4,9,52]. Beyond efficiency concerns, clinicians may also perceive such workflows as a threat to their autonomy or hesitate to disclose conflicting AI results to patients [52]. From a patient perspective, though, accuracy, accountability, transparency, and personal interaction seem to be valued above speed. Future implementation strategies should therefore balance clinical feasibility with patient needs.

As suggested by the qualitative results, trust may be strengthened when patients are informed that the AI system’s accuracy has been validated and that the physician is trained to use AI tools, understands their limitations, critically evaluates AI outputs, considers the patient’s individual circumstances beyond algorithmic recommendations, and uses AI to complement rather than replace their own clinical expertise. Emphasizing the physician’s central role in care, these assurances likely help address concerns about overreliance on AI. These considerations are especially meaningful against the lack of guidance on clinician disclosure and communication of hybrid medical decisions in current medical practice [78,80,81].

Although participants’ open-ended responses demonstrated that many were aware of the potential of AI-CDSS to improve clinical decisions, their quantitative and qualitative responses still appeared to have been influenced rather by perceived risks than by potential benefits of physician AI support. Hybrid decision-making was rarely evaluated more favorably than physician-only decisions. Nevertheless, all mean ratings were above the scale midpoint, and willingness to follow the medical advice remained relatively high across conditions. With increasing public familiarity with AI in health care, patient perceptions of hybrid medical decisions may improve. Educational efforts that explain the merit of AI-assisted medical decisions may help bridge the gap between technological advances and public acceptance, which is essential for realizing the full potential of AI-CDSS in clinical practice.

### Limitations

The following limitations should be considered. First, the use of vignettes involves a trade-off: although this approach enabled controlled manipulations of AI support type and timing, the scenarios remained hypothetical and lack the complexity of real clinical encounters. In particular, some parts of the vignettes were phrased rather generally, which could have created ambiguity, for example, about the respective contributions of the physician and the AI. Also, the vignettes did not specify the exact AI output or whether it confirmed the physician’s own assessment, although such agreement influences patient trust. Additionally, the observed effects may partly reflect how the physician introduced the AI support in the vignettes. While the vignettes were not designed to model a best-practice standard of disclosure and communication, the specific vignette wording could have lowered evaluations overall. More broadly, the experiments focused on only the type and timing of AI support, whereas other factors, such as physician communication and perceived AI expertise, may also shape patient trust. Despite the restricted ecological validity, however, the controlled design allowed isolating specific workflow features that have not yet been studied jointly, providing initial insight into their potential sociopsychological consequences.

Second, the findings’ generalizability is limited by the demographic composition of our samples. Both studies relied on relatively young, predominantly female, German online samples. Prior research suggests that younger individuals tend to be more accepting of AI than older individuals, while women tend to be less accepting than men [84-87]. Additionally, the samples consisted primarily of employees enrolled as part-time students and may therefore have had comparatively specific educational and digital backgrounds. We did not assess potentially relevant individual difference variables such as digital literacy, health literacy, or technological trust, and therefore cannot determine whether such factors moderated the observed effects. Also, the fit of the comparably younger sample to the ophthalmic context is limited. Glaucoma and diabetic retinopathy, described in 2 out of 4 scenarios, are more prevalent in older adults. Therefore, we also included consultation reasons and symptoms that could plausibly concern younger adults (eg, retinal detachment and preventive care). Overall, participants reported adequate levels of perceived vignette realism and ability to imagine the scenarios, suggesting that the materials were generally effective despite demographic restrictions. Nevertheless, because of the sample characteristics and hypothetical nature of vignette experiments, the findings are not directly generalizable to ophthalmic patient populations.

Third, willingness to follow the medical advice was assessed with only a single item, which may have been affected by a ceiling effect. Although such single-item measures are not uncommon in vignette-based studies on medical AI [22,30], they are less psychometrically robust than multi-item scales because they are more susceptible to random error and may be less sensitive to subtle between-condition differences [88,89]. In addition, the item captured a hypothetical behavioral intention, which may not accurately mirror real-world behavior [90].

Fourth, the exclusion rates of 8%‐15% per manipulation check suggest that several participants have missed key details of the vignettes. This may partly reflect both inattentive reading and the intentional subtlety of the manipulation: salient cues were avoided to reduce demand characteristics and a between-subjects manipulation of AI support was used so that participants would not directly compare different experimental conditions. Excluding participants who failed the manipulation checks may introduce survivorship bias, although intention-to-treat analyses produced a largely similar pattern of results. Similar or higher exclusion rates due to failed manipulation checks have also been reported in related vignette-based studies of AI in health care [29,41,54], indicating that such designs involve a trade-off between subtlety of manipulation and ease of participant discrimination.

Overall, the findings should not be interpreted as identifying the only or most influential determinants of trust in AI-supported medical decisions. Rather, the present findings are best understood as initial indications that patients may respond to *how* clinicians use and communicate AI-CDSS when encountering hybrid medical decisions.

### Future Research

Future research should examine more explicitly how physician communication and disclosure strategies shape patient responses to clinician-facing AI-CDSS. In particular, it would be valuable to disentangle the effects of AI use itself from the effects of how AI use and results are communicated to patients. Relatedly, future research should also examine whether responses to different forms of AI-assisted workflows vary as a function of individual differences, alignment of AI results with the physician’s assessment, and the specific health outcome (ie, favorable vs unfavorable). Additionally, studies in real clinical settings that obtain direct patient feedback on different AI-CDSS workflows, test the feasibility and desirability of sequential decision-making processes, and measure actual behavior rather than behavioral intentions would provide valuable insight. Ultimately, multistakeholder research is needed to ensure that AI-CDSS are not only technically effective but also trusted and accepted in clinical practice by all human stakeholders.

### Conclusions

AI-CDSS can be integrated into clinical decision-making in different ways, for example, depending on the type of support they provide and when they are used in the decision-making process. Understanding the patient perspective on these different forms of implementation is important, as patients’ trust in the resulting hybrid medical decisions contributes to the success of such tools in clinical practice. Across 2 vignette experiments, we examined how medical decisions made with different types and timings of clinician-facing AI support are perceived by members of the general public. We found that evaluations of hybrid clinical decisions depended on both the type of AI support and how the support was incorporated into the physician’s decision-making workflow. Decisions were evaluated less favorably when the physician used AI support that provided preliminary diagnostic suggestions than when the physician used AI support in the form of additional analyses or visualizations, but this difference was not observed when the physician first formed an independent clinical assessment before reviewing AI advice. By contrast, willingness to follow medical advice was relatively high, regardless of the specific implementation of AI support. Overall, the findings suggest that potential patients are sensitive to how physician-AI collaboration is structured, not merely to the presence of AI itself.

## Supplementary material

10.2196/93172 Multimedia Appendix 1 Pilot study and image pretest.

10.2196/93172 Multimedia Appendix 2 Results from additional analyses.

10.2196/93172 Multimedia Appendix 3 Vignettes.

10.2196/93172 Multimedia Appendix 4 Items.

10.2196/93172 Multimedia Appendix 5 Large language model prompts and suggested themes.

10.2196/93172 Checklist 1 GROVE checklist.

## Abbreviations

AI-CDSS: AI-based clinical decision support systems
EMM: estimated marginal mean
GROVE: Guideline for Reporting of Vignette Experiments
LMM: linear mixed-effects model
