# Supplementary material for: Effects of Type and Timing of Clinician-Facing AI Support on Patient Trust in Medical Consultations: 2 Vignette Experiments
Source: J Med Internet Res. 2026 Aug 21;28:e93172. doi: 10.2196/93172 (PMC13496942; doi:10.2196/93172)
Supplement: Supplementary file 1 [file jmir-v28-e93172-s001.docx]

**Multimedia Appendix 1**

**Pilot Study**

The pilot study served two main purposes: to obtain preliminary estimates for power calculations and to refine the study materials. The pilot was conducted between December 2024 and January 2025.

It used a design and procedure similar to those of the main studies. Participants (N=380), recruited via university subject pools and social media, were randomly assigned to one of four experimental conditions and read four vignettes presented in random order. The experimental conditions differed with regard to who made the medical decision and how the decision was derived. Compared with the main studies, the pilot study included one additional condition (“AI Only”). This condition was not retained in the main studies because fully autonomous AI-based medical decision-making was considered comparatively unrealistic in light of current clinical and regulatory practice.

The pilot vignettes covered the same four consultation types as the final vignettes used in the main studies (i.e., screening, emergency, follow-up, risk prediction). All outcome measures that were later used in the main studies were also completed by participants after each vignette in the pilot study. This allowed us to assess the internal consistency of the multi-item scales and to generate preliminary effect size estimates for power calculations. Participants also completed a manipulation check item. This item did not require participants to differentiate between the two hybrid conditions and was thus revised for the main studies to allow adequate discriminate between the two hybrid conditions.

At the end of the pilot study, participants were invited to provide open-ended feedback on how to improve the realism and immersiveness of the vignettes. Finally, demographic questions were moved to the end of the survey in the main studies to reduce participant fatigue during the vignette and outcome-assessment sections.

**Image Pretest**

A frequent recommendation in the pilot feedback was to include images in the vignettes to increase immersiveness. We therefore conducted a separate image pretest. A pool of candidate images was preselected for three scene categories corresponding to the general structure of the vignettes: (1) the entrance/waiting area, (2) the consultation room, and (3) the computer screen displaying the retinal image. Depending on the scene category, between 6 and 10 candidate images were initially considered.

Participants recruited via Prolific (N=50) rated all candidate images on valence and arousal, as assessed with one item each using 7-point Likert scales (Table S31).

**Table S31**. Means and SD of valence and arousal ratings for images used in the vignettes, grouped by scene category.

| Image used in respective vignette | | Valence | | | | Arousal | | |
| --- | --- | --- | --- | --- | --- | --- | --- | --- |
|  | | M | | SD | | M | | SD |
| **Entrance/waiting area** | | | | | | | | |
|  | Screening (diabetic retinopathy) | 3.48 | 0.79 | | 2.32 | | 1.24 | |
|  | Emergency (retinal detachment) | 3.70 | 1.04 | | 2.62 | | 1.21 | |
|  | Risk prediction (oculomics) | 3.60 | 0.90 | | 2.52 | | 1.09 | |
|  | Follow-up (glaucoma) | 3.62 | 0.90 | | 2.62 | | 1.21 | |
| **Consultation room** | | | | | | | | |
|  | Screening (diabetic retinopathy) | 3.62 | 0.88 | | 2.76 | | 1.53 | |
|  | Emergency (retinal detachment) | 3.80 | 0.76 | | 2.44 | | 1.25 | |
|  | Risk prediction (oculomics) | 3.74 | 0.80 | | 2.44 | | 1.34 | |
|  | Follow-up (glaucoma) | 3.90 | 0.79 | | 2.54 | | 1.42 | |
| **Computer screen** | | | | | | | | |
|  | Screening (diabetic retinopathy) | 3.50 | 1.05 | | 2.76 | | 1.38 | |
|  | Emergency (retinal detachment) | 3.40 | 0.88 | | 2.70 | | 1.33 | |
|  | Risk prediction (oculomics) | 3.50 | 1.00 | | 2.98 | | 1.55 | |
|  | Follow-up (glaucoma) | 3.66 | 1.04 | | 2.90 | | 1.50 | |

For each of the three scene categories, we selected four images (one for each vignette) that did not differ significantly in valence or arousal, based on one-way repeated-measures analyses of variance. The images were then assigned to one of the four vignettes, so that the depicted environment (e.g., clinic vs. private practice) best fit the respective vignette’s setting. The purpose of this pretest was to select image sets that were comparable in affective qualities across the four vignettes. These images were then used in the main studies.
