# Supplementary material for: Effects of Type and Timing of Clinician-Facing AI Support on Patient Trust in Medical Consultations: 2 Vignette Experiments
Source: J Med Internet Res. 2026 Aug 21;28:e93172. doi: 10.2196/93172 (PMC13496942; doi:10.2196/93172)
Supplement: Supplementary file 2 [file jmir-v28-e93172-s002.docx]

**Multimedia Appendix 2**

**Additional Analyses**

**Table S1.** Mean (SD) values of perceived realism of vignettes across studies.

| Vignette | Pilot  (N=380) | Study 1 (N=489) | Study 2 (N=570) |
| --- | --- | --- | --- |
| Screening | 5.26 (1.14) | 5.54 (1.06) | 5.52 (1.05) |
| Emergency | 5.37 (1.11) | 5.62 (1.00) | 5.72 (1.01) |
| Oculomics | 5.45 (1.07) | 5.50 (1.07) | 5.53 (1.08) |
| Follow-Up | 5.37 (1.08) | 5.58 (1.01) | 5.65 (0.99) |

**Table S2.** Means (SD) and ANOVA results from sentiment analyses in Study 1 (N=489).

| Sentiment | No AI | | Descriptive AI | | Diagnostic AI | | *F*(2, 486) | *P* value | $\eta^{2}$ |
| --- | --- | --- | --- | --- | --- | --- | --- | --- | --- |
|  | *M* | *SD* | *M* | *SD* | *M* | *SD* |  |  |  |
| Negative | 0.37 | 0.31 | 0.26 | 0.28 | 0.35 | 0.31 | 6.52 | **.002** | .03 |
| Neutral | 0.35 | 0.22 | 0.33 | 0.23 | 0.34 | 0.21 | 0.26 | .772 | < .001 |
| Positive | 0.28 | 0.29 | 0.41 | 0.34 | 0.32 | 0.30 | 7.81 | **< .001** | .03 |

*Note.* The possible range for sentiment scores was between 0 and 1. *P* values < .05 are shown in bold.

**Table S3.** *P* values from pairwise comparisons from sentiment analyses in Study 1 (N=489).

| Condition | | 1 | 2 | 3 |
| --- | --- | --- | --- | --- |
| 1 | No AI | **—** | **.002** | .435 |
| 2 | Descriptive AI | **<.001** | **—** | **.017** |
| 3 | Diagnostic AI | .289 | **.013** | **—** |

*Note*. Results for negative sentiment are presented above the diagonal and results for positive sentiment below the diagonal. The sentiment analysis was based on responses to the open-ended question Q2, which asked participants how they felt about the physician’s use of AI support in the vignettes (*Descriptive AI* and *Diagnostic* AI) or how they would feel if the physician had used AI support (*No AI*). Pairwise comparisons were tested with Bonferroni-Holm correction. *P* values < .05 are shown in bold.

**Table S4.** Bonferroni-Holm-adjusted *P* values for the omnibus tests across the four primary outcomes in Study 1 and Study 2.

| Outcome | Study 1:  condition | Study 2:  type × timing | Study 2:  condition |
| --- | --- | --- | --- |
| Trust in the medical decision | .004 | .022 | < .001 |
| Perceived provider trustworthiness | < .001 | .022 | < .001 |
| Perceived uniqueness neglect | .023 | .038 | < .001 |
| Willingness to follow the advice | .211 | .069 | < .001 |

*Note*. Holm-adjusted *P* values were calculated separately within each family of four primary omnibus tests. In Study 1, these were omnibus tests of condition. In Study 2, adjusted *P* values are reported separately for the 2×2 interaction models (comparing a model with the interaction term to a model with only main effects) and the all-conditions models.

**Table S5.** Pairwise contrasts of Estimated Marginal Means (EMMs) for Trust in the Medical Decisions in Study 1 (N=489).

| Contrast | Estimate | SE | *t*(486) | *P* value |
| --- | --- | --- | --- | --- |
| No AI – Descriptive AI | -0.09 | 0.11 | -0.86 | .390 |
| No AI – Diagnostic AI | 0.28 | 0.11 | 2.67 | **.016** |
| Descriptive AI – Diagnostic AI | 0.37 | 0.11 | 3.51 | **.002** |

*Note.* Pairwise contrasts were tested with Bonferroni-Holm correction. *P* values < .05 are shown in bold.

**Table S6.** Pairwise contrasts of EMMs for Perceived Provider Trustworthiness in Study 1 (N=489).

| Contrast | Estimate | SE | *t*(486) | *P* value |
| --- | --- | --- | --- | --- |
| No AI – Descriptive AI | -0.07 | 0.11 | -0.67 | .503 |
| No AI – Diagnostic AI | 0.40 | 0.11 | 3.81 | **<.001** |
| Descriptive AI – Diagnostic AI | 0.47 | 0.11 | 4.46 | **<.001** |

*Note.* Pairwise contrasts were tested with Bonferroni-Holm correction. *P* values < .05 are shown in bold.

**Table S7.** Pairwise contrasts of EMMs for Perceived Uniqueness Neglect in Study 1 (N=489).

| Contrast | Estimate | SE | *t*(486) | *P* value |
| --- | --- | --- | --- | --- |
| No AI – Descriptive AI | 0.03 | 0.12 | 0.26 | .795 |
| No AI – Diagnostic AI | -0.29 | 0.12 | -2.47 | **.028** |
| Descriptive AI – Diagnostic AI | -0.32 | 0.12 | -2.72 | **.021** |

*Note.* Pairwise contrasts were tested with Bonferroni-Holm correction. *P* values < .05 are shown in bold.

**Table S8.** Pairwise contrasts of EMMs for Willingness to Follow the Advice in Study 1 (N=489).

| Contrast | Estimate | SE | *t*(486) | *P* value |
| --- | --- | --- | --- | --- |
| No AI – Descriptive AI | -0.07 | 0.11 | -0.64 | .533 |
| No AI – Diagnostic AI | 0.12 | 0.11 | 1.11 | .533 |
| Descriptive AI – Diagnostic AI | 0.20 | 0.11 | 1.74 | .247 |

*Note.* Pairwise contrasts were tested with Bonferroni-Holm correction. *P* values < .05 are shown in bold.

**Table S9.** Intention-to-treat analysis for Linear Mixed Models (LMM) for each of the four outcome variables in Study 1 (N=584).

| Condition | | EMM [95% CI] | SE | Likelihood Ratio Test^a^ |
| --- | --- | --- | --- | --- |
| **Trust in the Medical Decisions** | | | | χ²(2) = 8.78, *P* = .012 |
|  | No AI | 5.18 [5.05, 5.30] | 0.06 |  |
|  | Descriptive AI | 5.29 [5.16, 5.43] | 0.07 |  |
|  | Diagnostic AI | 5.00 [4.86, 5.14] | 0.07 |  |
| **Perceived Provider Trustworthiness** | | | | χ²(2) = 17.41, *P* < .001 |
|  | No AI | 5.24 [5.11, 5.37] | 0.07 |  |
|  | Descriptive AI | 5.33 [5.19, 5.47] | 0.07 |  |
|  | Diagnostic AI | 4.94 [4.80, 5.07] | 0.07 |  |
| **Perceived Uniqueness Neglect** | | | | χ²(2) = 5.25, *P* = .073 |
|  | No AI | 3.06 [2.92, 3.20] | 0.07 |  |
|  | Descriptive AI | 3.02 [2.86, 3.17] | 0.08 |  |
|  | Diagnostic AI | 3.25 [3.10, 3.40] | 0.08 |  |
| **Willingness to Follow the Advice** | | | | χ²(2) = 2.23, *P* = .329 |
|  | No AI | 5.46 [5.32, 5.59] | 0.07 |  |
|  | Descriptive AI | 5.55 [5.40, 5.70] | 0.08 |  |
|  | Diagnostic AI | 5.39 [5.24, 5.54] | 0.07 |  |

^a^ Comparison of a model with *Condition* as fixed effects to a null model without this term.

**Table S10.** Intention-to-treat analysis for pairwise contrasts of EMMs for Trust in the Medical Decisions in Study 1 (N=584).

| Contrast | Estimate | SE | *t*(581) | *P* value |
| --- | --- | --- | --- | --- |
| No AI – Descriptive AI | -0.12 | 0.10 | -1.24 | .217 |
| No AI – Diagnostic AI | 0.18 | 0.10 | 1.83 | .136 |
| Descriptive AI – Diagnostic AI | 0.29 | 0.10 | 2.95 | **.010** |

***Note*.** Pairwise contrasts were tested with Bonferroni-Holm correction. *P* values < .05 are shown in bold.

**Table S11.** Intention-to-treat analysis for pairwise contrasts of EMMs for Perceived Provider Trustworthiness in Study 1 (N=584).

| contrast | estimate | SE | *t*(581) | *P* value |
| --- | --- | --- | --- | --- |
| No AI – Descriptive AI | -0.09 | 0.10 | -0.95 | .343 |
| No AI – Diagnostic AI | 0.31 | 0.10 | 3.19 | **.003** |
| Descriptive AI – Diagnostic AI | 0.40 | 0.10 | 3.98 | **< .001** |

***Note*.** Pairwise contrasts were tested with Bonferroni-Holm correction. *P* values < .05 are shown in bold.

**Table S12.** Intention-to-treat analysis for pairwise contrasts of EMMs for Perceived Uniqueness Neglect in Study 1 (N=584).

| contrast | estimate | SE | *t*(581) | *P* value |
| --- | --- | --- | --- | --- |
| No AI – Descriptive AI | 0.04 | 0.11 | 0.37 | .711 |
| No AI – Diagnostic AI | -0.19 | 0.11 | -1.84 | .134 |
| Descriptive AI – Diagnostic AI | -0.23 | 0.11 | -2.12 | .103 |

***Note*.** Pairwise contrasts were tested with Bonferroni-Holm correction.

**Table S13.** Intention-to-treat analysis for pairwise contrasts of EMMs for Willingness to Follow the Advice in Study 1 (N=584).

| contrast | estimate | SE | *t*(581) | *P* value |
| --- | --- | --- | --- | --- |
| No AI – Descriptive AI | -0.09 | 0.10 | -0.91 | .726 |
| No AI – Diagnostic AI | 0.06 | 0.10 | 0.63 | .726 |
| Descriptive AI – Diagnostic AI | 0.16 | 0.11 | 1.48 | .418 |

***Note*.** Pairwise contrasts were tested with Bonferroni-Holm correction.

**Table S14.** Exploratory analyses of Vignette main and interaction effects in Study 1 (N=489).

| Vignette | | EMM [95% CI] | SE | Likelihood Ratio Test of Main Effectᵃ | Likelihood Ratio Testᵃ of Interaction Effect^b^ |
| --- | --- | --- | --- | --- | --- |
| **Trust in the Medical Decisions** | | |  | χ²(3) = 40.36, *P* < .001 | χ²(6) = 4.35, *P* = .629 |
|  | Emergency | 5.32 [5.21, 5.43] | 0.06 |  |  |
|  | Follow-Up | 5.03 [4.92, 5.14] | 0.06 |  |  |
|  | Oculomics | 5.16 [5.05, 5.27] | 0.06 |  |  |
|  | Screening | 5.37 [5.25, 5.48] | 0.06 |  |  |
| **Perceived Provider Trustworthiness** | | |  | χ²(3) = 27.98, *P* < .001 | χ²(6) = 5.60, *P* = .469 |
|  | Emergency | 5.29 [5.19, 5.39] | 0.05 |  |  |
|  | Follow-Up | 5.09 [4.99, 5.19] | 0.05 |  |  |
|  | Oculomics | 5.21 [5.11, 5.31] | 0.05 |  |  |
|  | Screening | 5.29 [5.19, 5.39] | 0.05 |  |  |
| **Perceived Uniqueness Neglect** | | |  | χ²(3) = 27.59, *P* < .001 | χ²(6) = 7.95, *P* = .242 |
|  | Emergency | 2.94 [2.83, 3.06] | 0.06 |  |  |
|  | Follow-Up | 3.18 [3.07, 3.30] | 0.06 |  |  |
|  | Oculomics | 3.10 [2.98, 3.21] | 0.06 |  |  |
|  | Screening | 2.94 [2.83, 3.06] | 0.06 |  |  |
| **Willingness to Follow the Advice** | | |  | χ²(3) = 204.02, *P* < .001 | χ²(6) = 9.92, *P* = .128 |
|  | Emergency | 5.28 [5.15, 5.41] | 0.07 |  |  |
|  | Follow-Up | 5.02 [4.89, 5.14] | 0.07 |  |  |
|  | Oculomics | 5.99 [5.86, 6.12] | 0.07 |  |  |
|  | Screening | 5.84 [5.71, 5.97] | 0.07 |  |  |

^a^ Comparison of a model with *Vignette* as fixed effects to a model including only *Condition*.

^b^ Comparison of a model with the interaction term *Condition* x *Vignette* to a model including both main effects.

**Table S15.** Frequency of recurring themes in open-ended responses to Q1-Q3 in Study 1 (N=489).

| Themes | | n (%) |
| --- | --- | --- |
| **Influencing Factors (Q1)** | | |
|  | Aspects of the Medical Situation | 183 (37) |
|  | AI-System & Physician-AI-Interaction | 138 (28) |
|  | Trust & Relationship with the Physician | 123 (25) |
|  | Emotional & Personal Aspects | 66 (14) |
|  | Perception of the Healthcare System | 24 (5) |
| **Appraisal of AI Support (Q2)** | | |
|  | Physician-AI-Interaction | 237 (49) |
|  | Potential | 171 (35) |
|  | Concerns | 113 (23) |
|  | Neutral | 13 (3) |
| **Increasing Trust (Q3)** | | |
|  | Second Opinion | 153 (31) |
|  | Physician Demeanor & Communication | 137 (28) |
|  | Use of AI | 117 (24) |
|  | More Information about AI | 47 (10) |
|  | Nothing | 25 (5) |
|  | Recommendation by Others | 8 (2) |

**Table S16.** Means (SD) and ANOVA results from sentiment analyses in Study 2 (N=570).

| Sentiment | No AI | | Concurrent Descriptive AI | | Concurrent Diagnostic AI | | Sequential Descriptive AI | | Sequential Diagnostic AI | | *F*(4, 565) | *P* value | $\eta^{2}$ |
| --- | --- | --- | --- | --- | --- | --- | --- | --- | --- | --- | --- | --- | --- |
|  | *M* | *SD* | *M* | *SD* | *M* | *SD* | *M* | *SD* | *M* | *SD* |  |  |  |
| Negative | 0.43 | 0.34 | 0.24 | 0.27 | 0.42 | 0.32 | 0.23 | 0.26 | 0.35 | 0.33 | 10.55 | **<.001** | .07 |
| Neutral | 0.30 | 0.22 | 0.34 | 0.23 | 0.33 | 0.20 | 0.35 | 0.23 | 0.30 | 0.21 | 1.31 | .263 | <.001 |
| Positive | 0.27 | 0.29 | 0.42 | 0.32 | 0.26 | 0.28 | 0.42 | 0.31 | 0.35 | 0.33 | 7.21 | **<.001** | .05 |

*Note.* The possible range for sentiment scores was between 0 and 1. *P* values < .05 are shown in bold.

**Table S17.** *P* values from pairwise comparisons from sentiment analyses in Study 2 (N=570).

| Condition | | 1 | 2 | 3 | 4 | 5 |
| --- | --- | --- | --- | --- | --- | --- |
| 1 | No AI | **—** | **<.001** | >.999 | **<.001** | .131 |
| 2 | Concurrent Descriptive AI | **.003** | **—** | **<.001** | >.999 | .057 |
| 3 | Concurrent Diagnostic AI | >.999 | **.002** | **—** | **<.001** | .243 |
| 4 | Sequential Descriptive AI | **.001** | >.999 | **.001** | **—** | **.021** |
| 5 | Sequential Diagnostic AI | .167 | .358 | .104 | .358 | **—** |

*Note*. Results for negative sentiment are presented above the diagonal and results for positive sentiment below the diagonal. The sentiment analysis was based on responses to the open-ended question Q2, which asked participants how they felt about the physician’s use of AI support in the vignettes (*Descriptive AI* and *Diagnostic AI*) or how they would feel if the physician had used AI support (*No AI*). Pairwise comparisons were tested with Bonferroni-Holm correction. *P* values < .05 are shown in bold.

**Table S18.** Intention-to-treat analysis for each of the four outcome variables in Study 2 (N=612), using 2x2 LMMs.

| Condition | | Estimate | SE | *t*(608) | *P* value |
| --- | --- | --- | --- | --- | --- |
| **Trust in the Medical Decisions** | |  |  |  |  |
|  | Intercept | 5.38 | 0.07 |  |  |
|  | AI Support Typeᵃ | -0.49 | 0.11 | -4.61 | **< .001** |
|  | AI Support Timingᵇ | 0.10 | 0.10 | 0.95 | .342 |
|  | AI Support Type × AI Support Timing | 0.32 | 0.15 | 2.17 | **.030** |
| **Perceived Provider Trustworthiness** | |  |  |  |  |
|  | Intercept | 5.37 | 0.08 |  |  |
|  | AI Support Typeᵃ | -0.52 | 0.11 | -4.65 | **< .001** |
|  | AI Support Timingᵇ | 0.05 | 0.11 | 0.46 | .644 |
|  | AI Support Type × AI Support Timing | 0.41 | 0.16 | 2.66 | **.008** |
| **Perceived Uniqueness Neglect** | |  |  |  |  |
|  | Intercept | 2.94 | 0.09 |  |  |
|  | AI Support Typeᵃ | 0.52 | 0.12 | 4.22 | **< .001** |
|  | AI Support Timingᵇ | 0.01 | 0.12 | 0.06 | .954 |
|  | AI Support Type × AI Support Timing | -0.39 | 0.17 | -2.28 | **.023** |
| **Willingness to Follow the Advice** | |  |  |  |  |
|  | Intercept | 5.73 | 0.08 |  |  |
|  | AI Support Typeᵃ | -0.38 | 0.12 | -3.25 | **.001** |
|  | AI Support Timingᵇ | 0.07 | 0.11 | 0.63 | .531 |
|  | AI Support Type × AI Support Timing | 0.23 | 0.16 | 1.43 | .153 |

***Note*.** *P* values < .05 are shown in bold.

^a^ *Descriptive AI* served as reference category.

^b^ *Concurrent Timing* served as reference category.

**Table S19.** Exploratory analyses of Vignette main and interaction effects in Study 2 (N=440), using 2x2 LMMs.

| Vignette | | EMM [95% CI] | SE | Likelihood Ratio Test of Main Effectᵃ | Likelihood Ratio Testᵃ of Interaction Effect^b^ |
| --- | --- | --- | --- | --- | --- |
| **Trust in the Medical Decisions** | | |  | χ²(3) = 21.45, *P* < .001 | χ²(9) = 6.23, *P* = .717 |
|  | Emergency | 5.35 [5.24, 5.46] | 0.06 |  |  |
|  | Follow-Up | 5.18 [5.07, 5.29] | 0.06 |  |  |
|  | Oculomics | 5.27 [5.16, 5.38] | 0.06 |  |  |
|  | Screening | 5.46 [5.35, 5.57] | 0.06 |  |  |
| **Perceived Provider Trustworthiness** | | |  | χ²(3) = 14.53, *P* = .002 | χ²(9) = 6.44, *P* = .695 |
|  | Emergency | 5.39 [5.28, 5.49] | 0.05 |  |  |
|  | Follow-Up | 5.25 [5.15, 5.36] | 0.05 |  |  |
|  | Oculomics | 5.24 [5.14, 5.35] | 0.05 |  |  |
|  | Screening | 5.36 [5.25, 5.46] | 0.05 |  |  |
| **Perceived Uniqueness Neglect** | | |  | χ²(3) = 18.90, *P* < .001 | χ²(9) = 9.36, *P* = .405 |
|  | Emergency | 2.97 [2.84, 3.10] | 0.06 |  |  |
|  | Follow-Up | 3.09 [2.96, 3.21] | 0.06 |  |  |
|  | Oculomics | 3.20 [3.07, 3.33] | 0.06 |  |  |
|  | Screening | 2.96 [2.83, 3.09] | 0.06 |  |  |
| **Willingness to Follow the Advice** | | |  | χ²(3) = 227.72, *P* < .001 | χ²(9) = 9.17, *P* = .421 |
|  | Emergency | 5.36 [5.23, 5.49] | 0.07 |  |  |
|  | Follow-Up | 5.18 [5.05, 5.32] | 0.07 |  |  |
|  | Oculomics | 6.19 [6.05, 6.32] | 0.07 |  |  |
|  | Screening | 6.06 [5.93, 6.19] | 0.07 |  |  |

^a^ Comparison of a model with *Vignette* as fixed effects to a model including only the *Type* x *Timing* interaction term.

^b^ Comparison of a model with the interaction term *Type* x *Timing* x *Vignette* to a model including only the *Type* x *Timing* interaction term and the main effect of *Vignette*.

**Table S20.** Pairwise contrasts of EMMs for Trust in the Medical Decisions in Study 2 (N=570).

| Contrast | Estimate | SE | *t*(565) | *P* value |
| --- | --- | --- | --- | --- |
| No AI – Concurrent Descriptive AI | -0.33 | 0.12 | -2.76 | **.036** |
| No AI – Sequential Descriptive AI | -0.36 | 0.12 | -3.14 | **.012** |
| No AI – Concurrent Diagnostic AI | 0.31 | 0.12 | 2.65 | **.041** |
| No AI – Sequential Diagnostic AI | -0.18 | 0.11 | -1.58 | .449 |
| Concurrent Descriptive AI – Sequential Descriptive AI | -0.03 | 0.12 | -0.22 | .826 |
| Concurrent Descriptive AI – Concurrent Diagnostic AI | 0.64 | 0.13 | 5.09 | **<.001** |
| Concurrent Descriptive AI – Sequential Diagnostic AI | 0.16 | 0.12 | 1.29 | .449 |
| Sequential Descriptive AI – Concurrent Diagnostic AI | 0.67 | 0.12 | 5.57 | **<.001** |
| Sequential Descriptive AI – Sequential Diagnostic AI | 0.18 | 0.12 | 1.59 | .449 |
| Concurrent Diagnostic AI – Sequential Diagnostic AI | -0.48 | 0.12 | -4.13 | **<.001** |

***Note*.** Pairwise contrasts were tested with Bonferroni-Holm correction. *P* values < .05 are shown in bold.

**Table S21.** Pairwise contrasts of EMMs for Perceived Provider Trustworthiness in Study 2 (N=570).

| Contrast | Estimate | SE | *t*(565) | *P* value |
| --- | --- | --- | --- | --- |
| No AI – Concurrent Descriptive AI | -0.22 | 0.12 | -1.77 | .384 |
| No AI – Sequential Descriptive AI | -0.22 | 0.12 | -1.89 | .354 |
| No AI – Concurrent Diagnostic AI | 0.42 | 0.12 | 3.52 | **.003** |
| No AI – Sequential Diagnostic AI | -0.08 | 0.12 | -0.66 | > .999 |
| Concurrent Descriptive AI – Sequential Descriptive AI | -0.00 | 0.13 | -0.02 | > .999 |
| Concurrent Descriptive AI – Concurrent Diagnostic AI | 0.64 | 0.13 | 4.95 | **< .001** |
| Concurrent Descriptive AI – Sequential Diagnostic AI | 0.14 | 0.13 | 1.15 | .864 |
| Sequential Descriptive AI – Concurrent Diagnostic AI | 0.65 | 0.12 | 5.22 | **< .001** |
| Sequential Descriptive AI – Sequential Diagnostic AI | 0.15 | 0.12 | 1.24 | .864 |
| Concurrent Diagnostic AI – Sequential Diagnostic AI | -0.50 | 0.12 | -4.12 | **< .001** |

***Note*.** Pairwise contrasts were tested with Bonferroni-Holm correction. *P* values < .05 are shown in bold.

**Table S22.** Pairwise contrasts of EMMs for Perceived Uniqueness Neglect in Study 2 (N=570).

| Contrast | Estimate | SE | *t*(565) | *P* value |
| --- | --- | --- | --- | --- |
| No AI – Concurrent Descriptive AI | 0.14 | 0.14 | 0.96 | > .999 |
| No AI – Sequential Descriptive AI | 0.04 | 0.14 | 0.29 | > .999 |
| No AI – Concurrent Diagnostic AI | -0.53 | 0.14 | -3.84 | **< .001** |
| No AI – Sequential Diagnostic AI | -0.14 | 0.13 | -1.06 | > .999 |
| Concurrent Descriptive AI – Sequential Descriptive AI | -0.10 | 0.15 | -0.66 | > .999 |
| Concurrent Descriptive AI – Concurrent Diagnostic AI | -0.67 | 0.15 | -4.46 | **< .001** |
| Concurrent Descriptive AI – Sequential Diagnostic AI | -0.28 | 0.15 | -1.93 | .327 |
| Sequential Descriptive AI – Concurrent Diagnostic AI | -0.57 | 0.14 | -3.99 | **< .001** |
| Sequential Descriptive AI – Sequential Diagnostic AI | -0.18 | 0.14 | -1.32 | .946 |
| Concurrent Diagnostic AI – Sequential Diagnostic AI | 0.39 | 0.14 | 2.79 | **.038** |

***Note*.** Pairwise contrasts were tested with Bonferroni-Holm correction. *P* values < .05 are shown in bold.

**Table S23.** Pairwise contrasts of EMMs for Willingness to Follow the Advice in Study 2 (N=570).

| Contrast | Estimate | SE | *t*(565) | *P* value |
| --- | --- | --- | --- | --- |
| No AI – Concurrent Descriptive AI | -0.35 | 0.13 | -2.67 | .063 |
| No AI – Sequential Descriptive AI | -0.30 | 0.12 | -2.42 | .112 |
| No AI – Concurrent Diagnostic AI | 0.16 | 0.13 | 1.28 | .684 |
| No AI – Sequential Diagnostic AI | -0.13 | 0.12 | -1.06 | .684 |
| Concurrent Descriptive AI – Sequential Descriptive AI | 0.05 | 0.13 | 0.36 | .720 |
| Concurrent Descriptive AI – Concurrent Diagnostic AI | 0.51 | 0.14 | 3.74 | **.002** |
| Concurrent Descriptive AI – Sequential Diagnostic AI | 0.22 | 0.13 | 1.67 | .478 |
| Sequential Descriptive AI – Concurrent Diagnostic AI | 0.46 | 0.13 | 3.55 | **.004** |
| Sequential Descriptive AI – Sequential Diagnostic AI | 0.17 | 0.13 | 1.37 | .684 |
| Concurrent Diagnostic AI – Sequential Diagnostic AI | -0.29 | 0.13 | -2.28 | .138 |

***Note*.** Pairwise contrasts were tested with Bonferroni-Holm correction. *P* values < .05 are shown in bold.

**Table S24.** Intention-to-treat analysis for each of the four outcome variables in Study 2 (N=759), using all-conditions LMMs.

| Condition | | EMM [95% CI] | SE | Likelihood Ratio Testᵃ |
| --- | --- | --- | --- | --- |
| **Trust in the Medical Decisions** | |  |  | χ²(4) = 36.94, *P* < .001 |
|  | No AI | 5.17 [5.02, 5.32] | 0.08 |  |
|  | Concurrent Descriptive AI | 5.38 [5.23, 5.52] | 0.07 |  |
|  | Sequential Descriptive AI | 5.47 [5.33, 5.61] | 0.07 |  |
|  | Concurrent Diagnostic AI | 4.88 [4.74, 5.03] | 0.08 |  |
|  | Sequential Diagnostic AI | 5.31 [5.16, 5.45] | 0.08 |  |
| **Perceived Provider Trustworthiness** | |  |  | χ²(4) = 34.26, *P* < .001 |
|  | No AI | 5.27 [5.12, 5.43] | 0.08 |  |
|  | Concurrent Descriptive AI | 5.37 [5.22, 5.52] | 0.08 |  |
|  | Sequential Descriptive AI | 5.42 [5.27, 5.56] | 0.07 |  |
|  | Concurrent Diagnostic AI | 4.85 [4.69, 5.00] | 0.08 |  |
|  | Sequential Diagnostic AI | 5.31 [5.16, 5.46] | 0.08 |  |
| **Perceived Uniqueness Neglect** | |  |  | χ²(4) = 26.01, *P* < .001 |
|  | No AI | 2.97 [2.80, 3.14] | 0.09 |  |
|  | Concurrent Descriptive AI | 2.94 [2.77, 3.11] | 0.09 |  |
|  | Sequential Descriptive AI | 2.95 [2.79, 3.11] | 0.08 |  |
|  | Concurrent Diagnostic AI | 3.46 [3.29, 3.63] | 0.09 |  |
|  | Sequential Diagnostic AI | 3.08 [2.91, 3.25] | 0.09 |  |
| **Willingness to Follow the Advice** | |  |  | χ²(4) = 19.56, *P* < .001 |
|  | No AI | 5.52 [5.36, 5.68] | 0.08 |  |
|  | Concurrent Descriptive AI | 5.73 [5.57, 5.89] | 0.08 |  |
|  | Sequential Descriptive AI | 5.80 [5.65, 5.95] | 0.08 |  |
|  | Concurrent Diagnostic AI | 5.35 [5.19, 5.51] | 0.08 |  |
|  | Sequential Diagnostic AI | 5.66 [5.50, 5.82] | 0.08 |  |

^a^ Comparison of a model with *Condition* as fixed effects to a null model without this term.

**Table S25.** Intention-to-treat analysis for pairwise contrasts of EMMs for Trust in the Medical Decisions in Study 2 (N=759).

| Contrast | Estimate | SE | *z* ratio | *P* value |
| --- | --- | --- | --- | --- |
| No AI – Concurrent Descriptive AI | -0.20 | 0.11 | -1.92 | .275 |
| No AI – Sequential Descriptive AI | -0.30 | 0.10 | -2.91 | **.026** |
| No AI – Concurrent Diagnostic AI | 0.29 | 0.11 | 2.69 | **.043** |
| No AI – Sequential Diagnostic AI | -0.13 | 0.11 | -1.25 | .630 |
| Concurrent Descriptive AI – Sequential Descriptive AI | -0.10 | 0.10 | -0.95 | .680 |
| Concurrent Descriptive AI – Concurrent Diagnostic AI | 0.49 | 0.11 | 4.63 | **< .001** |
| Concurrent Descriptive AI – Sequential Diagnostic AI | 0.07 | 0.11 | 0.66 | .680 |
| Sequential Descriptive AI – Concurrent Diagnostic AI | 0.59 | 0.10 | 5.67 | **< .001** |
| Sequential Descriptive AI – Sequential Diagnostic AI | 0.17 | 0.10 | 1.62 | .421 |
| Concurrent Diagnostic AI – Sequential Diagnostic AI | -0.42 | 0.11 | -3.94 | **.001** |

*Note*. Tests are based on asymptotic (*z*) statistics (df = ∞). Bonferroni-Holm-adjusted *P* values are reported. *P* values < .05 are shown in bold.

**Table S26.** Intention-to-treat analysis for pairwise contrasts of EMMs for Perceived Provider Trustworthiness in Study 2 (N=759).

| Contrast | Estimate | SE | *z* ratio | *P* value |
| --- | --- | --- | --- | --- |
| No AI – Concurrent Descriptive AI | -0.09 | 0.11 | -0.85 | > .999 |
| No AI – Sequential Descriptive AI | -0.14 | 0.11 | -1.33 | > .999 |
| No AI – Concurrent Diagnostic AI | 0.43 | 0.11 | 3.87 | **.001** |
| No AI – Sequential Diagnostic AI | -0.04 | 0.11 | -0.34 | > .999 |
| Concurrent Descriptive AI – Sequential Descriptive AI | -0.05 | 0.11 | -0.47 | > .999 |
| Concurrent Descriptive AI – Concurrent Diagnostic AI | 0.52 | 0.11 | 4.75 | **< .001** |
| Concurrent Descriptive AI – Sequential Diagnostic AI | 0.05 | 0.11 | 0.50 | > .999 |
| Sequential Descriptive AI – Concurrent Diagnostic AI | 0.57 | 0.11 | 5.32 | **< .001** |
| Sequential Descriptive AI – Sequential Diagnostic AI | 0.11 | 0.11 | 0.98 | > .999 |
| Concurrent Diagnostic AI – Sequential Diagnostic AI | -0.46 | 0.11 | -4.22 | **< .001** |

*Note*. Tests are based on asymptotic (*z*) statistics (df = ∞). Bonferroni-Holm-adjusted *P* values are reported. *P* values < .05 are shown in bold.

**Table S27.** Intention-to-treat analysis for pairwise contrasts of EMMs for Perceived Uniqueness Neglect in Study 2 (N=759).

| Contrast | Estimate | SE | *z* ratio | *P* value |
| --- | --- | --- | --- | --- |
| No AI – Concurrent Descriptive AI | 0.03 | 0.12 | 0.26 | > .999 |
| No AI – Sequential Descriptive AI | 0.02 | 0.12 | 0.20 | > .999 |
| No AI – Concurrent Diagnostic AI | -0.49 | 0.12 | -4.01 | **< .001** |
| No AI – Sequential Diagnostic AI | -0.10 | 0.12 | -0.86 | > .999 |
| Concurrent Descriptive AI – Sequential Descriptive AI | -0.01 | 0.12 | -0.06 | > .999 |
| Concurrent Descriptive AI – Concurrent Diagnostic AI | -0.52 | 0.12 | -4.29 | **< .001** |
| Concurrent Descriptive AI – Sequential Diagnostic AI | -0.14 | 0.12 | -1.12 | > .999 |
| Sequential Descriptive AI – Concurrent Diagnostic AI | -0.51 | 0.12 | -4.33 | **< .001** |
| Sequential Descriptive AI – Sequential Diagnostic AI | -0.13 | 0.12 | -1.09 | > .999 |
| Concurrent Diagnostic AI – Sequential Diagnostic AI | 0.39 | 0.12 | 3.15 | **.011** |

*Note*. Tests are based on asymptotic (*z*) statistics (df = ∞). Bonferroni-Holm-adjusted *P* values are reported. *P* values < .05 are shown in bold.

**Table S28.** Intention-to-treat analysis for pairwise contrasts of EMMs for Willingness to Follow the Advice in Study 2 (N=759).

| Contrast | Estimate | SE | *z* ratio | *P* value |
| --- | --- | --- | --- | --- |
| No AI – Concurrent Descriptive AI | -0.21 | 0.12 | -1.81 | .422 |
| No AI – Sequential Descriptive AI | -0.28 | 0.11 | -2.48 | .092 |
| No AI – Concurrent Diagnostic AI | 0.17 | 0.12 | 1.47 | .704 |
| No AI – Sequential Diagnostic AI | -0.13 | 0.12 | -1.15 | .778 |
| Concurrent Descriptive AI – Sequential Descriptive AI | -0.07 | 0.11 | -0.64 | > .999 |
| Concurrent Descriptive AI – Concurrent Diagnostic AI | 0.38 | 0.12 | 3.29 | **.009** |
| Concurrent Descriptive AI – Sequential Diagnostic AI | 0.07 | 0.12 | 0.65 | > .999 |
| Sequential Descriptive AI – Concurrent Diagnostic AI | 0.45 | 0.11 | 3.99 | **.001** |
| Sequential Descriptive AI – Sequential Diagnostic AI | 0.15 | 0.11 | 1.30 | .778 |
| Concurrent Diagnostic AI – Sequential Diagnostic AI | -0.30 | 0.12 | -2.63 | .069 |

*Note*. Tests are based on asymptotic (*z*) statistics (df = ∞). Bonferroni-Holm-adjusted *P* values are reported. *P* values < .05 are shown in bold.

**Table S29.** Exploratory analyses of Vignette main and interaction effects in Study 2, using all-conditions LMMs (N=570).

| Vignette | | EMM [95% CI] | SE | Likelihood Ratio Test of Main Effectᵃ | Likelihood Ratio Testᵃ of Interaction Effect^b^ |
| --- | --- | --- | --- | --- | --- |
| **Trust in the Medical Decisions** | | |  | χ²(3) = 41.86, *P* < .001 | χ²(12) = 18.37, *P* = .105 |
|  | Emergency | 5.37 [5.27, 5.47] | 0.05 |  |  |
|  | Follow-Up | 5.12 [5.02, 5.22] | 0.05 |  |  |
|  | Oculomics | 5.21 [5.11, 5.31] | 0.05 |  |  |
|  | Screening | 5.45 [5.35, 5.55] | 0.05 |  |  |
| **Perceived Provider Trustworthiness** | | |  | χ²(3) = 38.74, *P* < .001 | χ²(12) = 20.91, *P* = .052 |
|  | Emergency | 5.41 [5.32, 5.50] | 0.05 |  |  |
|  | Follow-Up | 5.22 [5.12, 5.31] | 0.05 |  |  |
|  | Oculomics | 5.21 [5.12, 5.30] | 0.05 |  |  |
|  | Screening | 5.38 [5.29, 5.47] | 0.05 |  |  |
| **Perceived Uniqueness Neglect** | | |  | χ²(3) = 31.57, *P* < .001 | χ²(12) = 11.15, *P* = .517 |
|  | Emergency | 2.93 [2.82, 3.04] | 0.06 |  |  |
|  | Follow-Up | 3.06 [2.95, 3.17] | 0.06 |  |  |
|  | Oculomics | 3.20 [3.09, 3.31] | 0.06 |  |  |
|  | Screening | 2.93 [2.82, 3.04] | 0.06 |  |  |
| **Willingness to Follow the Advice** | | |  | χ²(3) = 287.11, *P* < .001 | χ²(12) = 22.25, *P* = .035 |
|  | Emergency | 5.39 [5.27, 5.50] | 0.06 |  |  |
|  | Follow-Up | 5.09 [4.97, 5.20] | 0.06 |  |  |
|  | Oculomics | 6.13 [6.02, 6.25] | 0.06 |  |  |
|  | Screening | 6.06 [5.94, 6.18] | 0.06 |  |  |

^a^ Comparison of a model with *Vignette* as fixed effects to a model including only *Condition*.

^b^ Comparison of a model with the interaction term *Condition* x *Vignette* to a model including both main effects.

**Table S30.** Frequency of recurring themes in open-ended responses to Q2 and Q3 in Study 2 (N=570).

| Themes | | n (%) |
| --- | --- | --- |
| **Appraisal of AI Support (Q2)** | | |
|  | Physician-AI-Interaction | 258 (45) |
|  | Potential | 188 (33) |
|  | Concerns | 134 (24) |
|  | Neutral | 16 (3) |
| **Increasing Trust (Q3)** | | |
|  | Use of AI | 162 (28) |
|  | Physician Demeanor & Communication | 137 (24) |
|  | Second Opinion | 133 (23) |
|  | More Information about AI | 98 (17) |
|  | Nothing | 45 (8) |
|  | Recommendation by Others | 7 (1) |
