# Supplementary material for: Effects of Type and Timing of Clinician-Facing AI Support on Patient Trust in Medical Consultations: 2 Vignette Experiments
Source: J Med Internet Res. 2026 Aug 21;28:e93172. doi: 10.2196/93172 (PMC13496942; doi:10.2196/93172)
Supplement: Supplementary file 3 [file jmir-v28-e93172-s003.docx]

**Multimedia Appendix 3**

**Vignettes (translated into English)**

**Study 1**

Note: All vignette images depicting people were licensed stock images; the individuals shown in these images were not study participants, patients, or members of the research team. Retinal images were taken from publicly available datasets: INSPIRE-AVR [1], HRF [2], OCTID [3], and REFUGE2 [4].

1.	Niemeijer M, Xu X, Dumitrescu AV, Gupta P, Van Ginneken B, Folk JC, Abramoff MD. Automated measurement of the arteriolar-to-venular width ratio in digital color fundus photographs. IEEE Trans Med Imaging 2011;30(11):1941–1950. doi: 10.1109/TMI.2011.2159619

2.	Budai A, Bock R, Maier A, Hornegger J, Michelson G. Robust vessel segmentation in fundus images. Int J Biomed Imaging 2013;2013:154860. doi: 10.1155/2013/154860

3.	Gholami P, Roy P, Parthasarathy MK, Lakshminarayanan V. OCTID: Optical coherence tomography image database. Comput Electr Eng 2020;81:106532. doi: 10.1016/j.compeleceng.2019.106532

4.	Fang H, Li F, Wu J, Fu H, Sun X, Son J, Yu S, Zhang M, Yuan C, Bian C, Lei B, Zhao B, Xu X, Li S, Fumero F, Sigut J, Almubarak H, Bazi Y, Guo Y, Zhou Y, Baid U, Innani S, Guo T, Yang J, Orlando JI, Bogunovic H, Zhang X, Xu Y. REFUGE2 Challenge: A Treasure Trove for Multi-Dimension Analysis and Evaluation in Glaucoma Screening. arXiv; 2022. doi: 10.48550/arXiv.2202.08994

***Screening***

**Please now imagine the following situation:**

You have had type II diabetes for several years. As this increases your risk of developing complications, you undergo regular check-ups. One of the possible complications is diabetic retinopathy. This is an eye disease that can impair vision and lead to blindness.

Even though you have not noticed any symptoms so far, you would like to have the recommended routine eye check-up to detect any possible diseases.

Since your usual ophthalmologist's office is temporarily closed, you visit a substitute practice and, after registering at the reception desk, are asked to take a seat in the waiting area.

A medical assistant calls your name and shows you to an examination room. The medical assistant asks you a few questions about your diabetes and asks you if you have any vision problems and what medications you are currently taking.

She then performs an eye test and takes a photo of your retina using a fundus camera. Such images can reveal, for example, bleeding or new blood vessel formation, which are signs of diabetic retinopathy.

One of the ophthalmologists working there then enters the consultation room. He reviews the results of the prior examination and examines your eyes using an ophthalmoscope.

Then he pulls up the fundus photo on his computer.

No AI

The doctor informs you that when evaluating the fundus photo, he pays attention to the structural characteristics of the retina, such as the shape of the blood vessels and certain color patterns of the retina.

The ophthalmologist then briefly summarizes all the findings and diagnoses mild to moderate diabetic retinopathy. He recommends that you make an earlier follow-up appointment for more comprehensive diagnostics to determine whether laser treatment is necessary.

Descriptive AI

The doctor informs you that he is using a computer program based on artificial intelligence (AI) to assist in the evaluation of the fundus photo.

The AI program measures the structural characteristics of the retina, such as the shape of the blood vessels and certain color patterns of the retina. However, the AI program is not used to suggest a diagnosis to the doctor, but serves purely as visual support in evaluating the photo.

The ophthalmologist then briefly summarizes all the findings and diagnoses mild to moderate diabetic retinopathy. He recommends that you make an earlier follow-up appointment for more comprehensive diagnostics to determine whether laser treatment is necessary.

Diagnostic AI

The doctor informs you that he is using a computer program based on artificial intelligence (AI) to assist in the evaluation of the fundus photo and in making a diagnosis.

The AI program measures the structural characteristics of the retina, such as the shape of the blood vessels and certain color patterns of the retina. The AI program also suggests a preliminary diagnosis to the doctor. The AI program thus provides the doctor with both visual support in evaluating the photo and assistance in making the diagnosis.

The ophthalmologist then briefly summarizes all the findings and diagnoses mild to moderate diabetic retinopathy. He recommends that you make an earlier follow-up appointment for more comprehensive diagnostics to determine whether laser treatment is necessary.

***Emergency***

**Please now imagine the following situation:**

You suddenly notice dark spots in your vision and can only see blurry images. You therefore go to the emergency room of the nearest eye clinic.

At the reception, you describe your symptoms, and the medical assistant asks you further questions about your symptoms and medical history. While the on-call ophthalmologist is being informed, you are taken directly to an examination room.

There, a medical assistant already begins with some initial examinations, including a vision test and a measurement of your intraocular pressure. She also uses an optical coherence tomography device to take 3D images of your retina, which may help to identify possible causes of the vision problems.

Afterwards, the on-call ophthalmologist enters the room. He reviews the results of the prior examinations and examines your eyes using an ophthalmoscope.

He then pulls up the images of your retina on his computer.

No AI

The doctor informs you that when evaluating the retinal images, he pays attention to the different layers of the retina, which appear with varying brightness in the images. He looks for areas where these retinal layers show unusual size or brightness.

The ophthalmologist then briefly summarizes all the findings and diagnoses retinal detachment. In a retinal detachment, the retina separates from the underlying layer of the inner wall of the eye, which can lead to sudden vision problems or blindness if not treated promptly. He recommends immediate surgery to preserve as much of your vision as possible.

Descriptive AI

The doctor informs you that he is using a computer program based on artificial intelligence (AI) to support in the evaluation of the retinal images.

The AI program measures the different layers of the retina, which appear with varying brightness in the images. The AI program looks for areas where these retinal layers show unusual size or brightness. However, the AI program is not used to suggest a diagnosis to the doctor, but serves purely as visual support in evaluating the images.

The ophthalmologist then briefly summarizes all the findings and diagnoses retinal detachment. In a retinal detachment, the retina separates from the underlying layer of the inner wall of the eye, which can lead to sudden vision problems or blindness if not treated promptly. He recommends immediate surgery to preserve as much of your vision as possible.

Diagnostic AI

The doctor informs you that he uses a computer program based on artificial intelligence (AI) to support in the evaluation of the retinal images and in making a diagnosis.

The AI program measures the different layers of the retina, which appear with varying brightness in the images. The AI program looks for areas where these retinal layers show unusual size or brightness. The AI program also suggests a preliminary diagnosis to the doctor. The AI program thus provides the doctor with both visual support in evaluating the images and assistance in making the diagnosis.

The ophthalmologist then briefly summarizes all the findings and diagnoses retinal detachment. In a retinal detachment, the retina separates from the underlying layer of the inner wall of the eye, which can lead to sudden vision problems or blindness if not treated promptly. He recommends immediate surgery to preserve as much of your vision as possible.

***Oculomics***

**Please now imagine the following situation:**

You recently received an official information brochure explaining the importance of preventive health check-ups. With the help of so-called health check-ups, personal risk factors for certain diseases can be identified and certain diseases can be detected at an early stage. You have decided to have such a health check-up done. Therefore, you visit a health center that specializes in this type of check-up. At the reception desk, you receive various documents and are asked to take a seat in the waiting area.

The documents inform you that in this health center a procedure is used for such check-ups in which the eyes are examined for certain characteristics (e.g., the vessels and tissue of the retina). These retinal characteristics can provide information about the risk of cardiovascular and neurological diseases.

After spending some time in the waiting area, a medical assistant calls you into the examination room and records your medical history and relevant health data. She then takes a photo of your retina using a fundus camera.

After the fundus photo has been taken, you are asked to take a seat in the waiting area again.

You are then called back into an examination room by a medical assistant, where one of the ophthalmologists working there pulls up the fundus photo of your retina on his computer.

No AI

The doctor informs you that when evaluating the fundus photo, he looks at the characteristics of the retina, such as the thickness of the blood vessels, and pays attention to certain features that may indicate whether or not a disease is present.

The ophthalmologist then briefly summarizes all the information. He informs you that you have an increased risk of stroke and recommends a more detailed examination by a neurologist.

Descriptive AI

The doctor informs you that he is using a computer program based on artificial intelligence (AI) to assist in evaluating the fundus photo.

The AI program measures the characteristics of the retina, such as the thickness of the blood vessels, and marks certain features that may indicate whether or not a disease is present. However, the AI program is not used to suggest a diagnosis to the doctor, but serves purely as visual support in evaluating the images.

The ophthalmologist then briefly summarizes all the information. He informs you that you have an increased risk of stroke and recommends a more detailed examination by a neurologist.

Diagnostic AI

The doctor informs you that he is using a computer program based on artificial intelligence (AI) to assist in evaluating the fundus photo and in making a diagnosis.

The AI program measures the characteristics of the retina, such as the thickness of the blood vessels, and marks certain features in the images that may indicate whether or not a disease is present. The AI program also suggests a preliminary diagnosis to the doctor. The AI program thus provides the doctor with both visual support in evaluating the photo and assistance in making the diagnosis.

The ophthalmologist then briefly summarizes all the information. He informs you that you have an increased risk of stroke and recommends a more detailed examination by a neurologist.

***Follow-up***

**Please now imagine the following situation:**

Two years ago, you were diagnosed with glaucoma affecting your left eye. This is an eye disease that can be caused by abnormal intraocular pressure, for example, and permanently damages the optic nerve. It causes "blind spots" in your vision. If the disease progresses, the affected eye can go blind. Medicated eye drops can often prevent the disease from worsening.

Now it's time for one of your regular follow-ups. Since your regular eye doctor is on vacation for an extended period, you visit a substitute practice. Once there, you are asked to take a seat in the waiting area.

After a while, a medical assistant calls you into a consultation room and takes various measurements, including intraocular pressure and visual field measurements (perimetry).

She also takes a photo of your retina using a fundus camera, which can provide information about possible changes in your glaucoma.

After a short wait, the ophthalmologist enters the consultation room. He asks you if you have any symptoms and if you are taking your medication regularly.
He briefly reviews the results of the prior examination and calls up the fundus photo of your retina on his computer.

No AI

The doctor informs you that when evaluating the fundus photo, he pays attention to certain retinal structures, such as the structure of the optic nerve head. He also compares the current images with previous ones.

The ophthalmologist then briefly summarizes all the information. He informs you that despite the medication, the glaucoma has worsened and your "blind spots" have enlarged. The doctor recommends that you undergo laser treatment.

Descriptive AI

The doctor informs you that he is using a computer program based on artificial intelligence (AI) to assist in evaluating the fundus photo.

The AI program measures certain retinal structures, such as the structure of the optic nerve head. It also creates a trend by comparing the current images with previous ones. However, the AI program is not used to suggest a diagnosis to the doctor, but serves purely as visual support in evaluating the photo.

The ophthalmologist then briefly summarizes all the information. He informs you that despite the medication, the glaucoma has worsened and your "blind spots" have enlarged. The doctor recommends that you undergo laser treatment.

Diagnostic AI

The doctor informs you that he is using a computer program based on artificial intelligence (AI) to assist in evaluating the fundus photo and making a diagnosis.

The AI program measures certain retinal structures, such as the structure of the optic nerve head. It also creates a trend by comparing the current images with previous ones. The AI program also suggests a preliminary diagnosis to the doctor. The AI program thus provides the doctor with both visual support in evaluating the photo and assistance in making the diagnosis.

The ophthalmologist then briefly summarizes all the information. He informs you that despite the medication, the glaucoma has worsened and your "blind spots" have enlarged. The doctor recommends that you undergo laser treatment.

**Study 2**

***Screening***

**Please now imagine the following situation:**

You have had type II diabetes for several years. As this increases your risk of developing complications, you undergo regular check-ups. One of the possible complications is diabetic retinopathy. This is an eye disease that can impair vision and lead to blindness.

Even though you have not noticed any symptoms so far, you would like to have the recommended routine eye check-up to detect any possible diseases.

Since your usual ophthalmologist's office is temporarily closed, you visit a substitute practice and, after registering at the reception desk, are asked to take a seat in the waiting area.

A medical assistant calls your name and shows you to an examination room. The medical assistant asks you a few questions about your diabetes and asks you if you have any vision problems and what medications you are currently taking.

She then performs an eye test and takes a photo of your retina using a fundus camera. Such images can reveal, for example, bleeding or new blood vessel formation, which are signs of diabetic retinopathy.

One of the ophthalmologists working there then enters the consultation room. He reviews the results of the prior examination and examines your eyes using an ophthalmoscope.

Then he calls up the fundus photo on his computer and examines it.

No AI

The ophthalmologist then summarizes the results of the examination for you and shares his interpretation of the fundus photo.

He diagnoses mild to moderate diabetic retinopathy and recommends that you make an earlier follow-up appointment for more comprehensive diagnostics to determine whether laser treatment is necessary.

Concurrent Descriptive AI

The ophthalmologist informs you that he uses a computer program based on artificial intelligence (AI) as a visual aid in evaluating the fundus photograph. This AI program measures the structural characteristics of the retina, such as the shape of the blood vessels and certain color patterns of the retina.

However, the AI program is not used to suggest a diagnosis to the doctor, but serves purely as visual support in evaluating the photo.

The doctor then summarizes all the findings for you.

He diagnoses mild to moderate diabetic retinopathy and recommends that you make an earlier follow-up appointment for more comprehensive diagnostics to determine whether laser treatment is necessary.

Sequential Descriptive AI

The ophthalmologist then summarizes the results of the examination for you and shares his interpretation of the fundus photo.

He then informs you that he will now use a computer program based on artificial intelligence (AI) to obtain an additional evaluation of the fundus photograph. This AI program measures the structural characteristics of the retina, such as the shape of the blood vessels and certain color patterns of the retina.

However, the AI program is not used to suggest a diagnosis to the doctor, but serves purely as visual support in evaluating the photo.

The doctor informs you of the results of the AI program and describes whether they correspond to his own assessment and how they are incorporated into the overall evaluation.

He diagnoses mild to moderate diabetic retinopathy and recommends that you make an earlier follow-up appointment for more comprehensive diagnostics to determine whether laser treatment is necessary.

Concurrent Diagnostic AI

The ophthalmologist informs you that he is using a computer program based on artificial intelligence (AI) for visual and diagnostic support in evaluating the fundus photo. This AI program measures the structural characteristics of the retina, such as the shape of the blood vessels and certain color patterns of the retina.

The AI program also suggests a preliminary diagnosis to the doctor. Thus, the AI program provides the doctor with both visual support in evaluating the photo and support in making the diagnosis.

The doctor then summarizes all the findings for you.

He diagnoses mild to moderate diabetic retinopathy and recommends that you make an earlier follow-up appointment for more comprehensive diagnostics to determine whether laser treatment is necessary.

Sequential Diagnostic AI

The ophthalmologist then summarizes the results of the examination for you and shares his interpretation of the fundus photo.

He then informs you that he will now use a computer program based on artificial intelligence (AI) to obtain an additional evaluation of the fundus photo. This AI program measures the structural properties of the retina, such as the shape of the blood vessels and certain color patterns of the retina.

The AI program also suggests a preliminary diagnosis to the doctor. Thus, the AI program provides the doctor with both visual support in evaluating the photo and assistance in making the diagnosis.

The doctor informs you of the results of the AI program and describes whether they correspond to his own assessment and how they are incorporated into the overall evaluation.

He diagnoses mild to moderate diabetic retinopathy and recommends that you make an earlier follow-up appointment for more comprehensive diagnostics to determine whether laser treatment is necessary.

***Emergency***

**Please now imagine the following situation:**

You suddenly notice dark spots in front of your eyes and can only see blurry images. You therefore go to the emergency room of the nearest eye clinic.

You describe your symptoms at the reception desk and the medical assistant asks you further questions about your symptoms and medical history. While the on-call ophthalmologist is being informed, you are taken directly to an examination room.

There, a medical assistant performs initial examinations, including a vision test and an intraocular pressure measurement. She also uses an optical coherence tomography device to take 3D images of your retina, which may help to identify possible causes of your vision problems. Such images can reveal abnormalities in the different layers of the retina, which are displayed in varying degrees of brightness.

Afterwards, the on-call ophthalmologist enters the room. He reviews the results of the prior examinations and examines your eyes using an ophthalmoscope.

He then pulls up the images of your retina on his computer and scrolls through the cross-sectional images of the 3D scans.

No AI

The ophthalmologist then summarizes all the findings for you and shares his interpretation of the retinal images.

He diagnoses retinal detachment. In a detached retina, the retina separates from the underlying layer of the inner wall of the eye, which can lead to sudden vision problems or blindness if not treated promptly. He recommends immediate surgery to preserve your vision as much as possible.

Concurrent Descriptive AI

The ophthalmologist informs you that he is using a computer program based on artificial intelligence (AI) to assist him in evaluating the retinal images. This AI program measures the different layers of the retina and searches for areas where these retinal layers have a noticeable size or brightness.

However, the AI program is not used to suggest a diagnosis to the doctor, but serves purely as visual support in evaluating the images.

The doctor then summarizes all the findings for you.

He diagnoses a retinal detachment. In a retinal detachment, the retina separates from the underlying layer of the inner wall of the eye, which can lead to sudden vision problems or blindness if not treated promptly. He recommends immediate surgery to preserve your vision as much as possible.

Sequential Descriptive AI

The ophthalmologist then summarizes all the findings for you and shares his interpretation of the retinal images.

He then informs you that he will now use a computer program based on artificial intelligence (AI) to obtain an additional evaluation of the retinal images. This AI program measures the different layers of the retina and searches for areas where these retinal layers have a noticeable size or brightness.

However, the AI program is not used to suggest a diagnosis to the doctor, but serves purely as visual support in evaluating the images.

The doctor informs you of the results of the AI program and describes whether they correspond to his own assessment and how they are incorporated into the overall evaluation.

He diagnoses a retinal detachment. In a retinal detachment, the retina separates from the underlying layer of the inner wall of the eye, which can lead to sudden vision problems or blindness if not treated promptly. He recommends immediate surgery to preserve your vision as much as possible.

Concurrent Diagnostic AI

The ophthalmologist informs you that he is using a computer program based on artificial intelligence (AI) for visual and diagnostic support in evaluating the retinal images. This AI program measures the different layers of the retina and searches for areas where these retinal layers have a noticeable size or brightness.

The AI program also suggests a preliminary diagnosis to the doctor. The AI program thus provides the doctor with both visual support in evaluating the photo and assistance in making the diagnosis.

The doctor then summarizes all the findings for you.

He diagnoses a retinal detachment. In a retinal detachment, the retina separates from the underlying layer of the inner wall of the eye, which can lead to sudden vision problems or blindness if not treated promptly. He recommends immediate surgery to preserve your vision as much as possible.

Sequential Diagnostic AI

The ophthalmologist then summarizes all the findings for you and shares his interpretation of the retinal images.

He then informs you that he will now use a computer program based on artificial intelligence (AI) to obtain an additional evaluation of the retinal images. This AI program measures the different layers of the retina and searches for areas where these retinal layers have a noticeable size or brightness.

The AI program also suggests a preliminary diagnosis to the doctor. The AI program thus offers the doctor both visual support in evaluating the images and assistance in making the diagnosis.

The doctor informs you of the results of the AI program and describes whether they correspond to his own assessment and how they are incorporated into the overall evaluation.

He diagnoses a retinal detachment. In a retinal detachment, the retina separates from the underlying layer of the inner wall of the eye, which can lead to sudden vision problems or blindness if not treated promptly. He recommends immediate surgery to preserve your vision as much as possible.

***Oculomics***

**Please now imagine the following situation:**

You recently received an official information brochure explaining the importance of preventive health check-ups. With the help of so-called health check-ups, diseases and personal risk factors can be identified at an early stage. You have decided to have such a health check-up. Therefore, you visit a health center that specializes in this type of check-up. At the reception desk, you receive various documents and are asked to take a seat in the waiting area.

The documents inform you that in this health center a procedure is used for such check-ups that examines the eyes for certain characteristics (e.g., the vessels and tissue of the retina). These retinal characteristics can provide information about the risk of cardiovascular and neurological diseases.

After spending some time in the waiting area, a medical assistant calls you into the examination room and records your medical history and relevant health data. She then uses a fundus camera to take a photo of your retina, which can be used to identify risk-relevant retinal features.

After the fundus photo has been taken, you are asked to take a seat in the waiting area again.

A medical assistant then calls you back into the examination room. One of the ophthalmologists working there pulls up the fundus photo of your retina on his computer and examines it.

No AI

The ophthalmologist then shares his interpretation of the fundus photo with you.

He informs you that you have an increased risk of stroke and recommends a more detailed examination by a neurologist.

Concurrent Descriptive AI

The ophthalmologist informs you that he uses a computer program based on artificial intelligence (AI) as a visual aid in evaluating the fundus photo. This AI program measures the characteristics of the retina, such as the thickness of the blood vessels, and marks certain features that may indicate whether or not there is an increased risk of disease.

However, the AI program is not used to suggest a diagnosis to the doctor, but serves purely as visual support in evaluating the photo.

The doctor then summarizes the findings for you.

He informs you that you have an increased risk of stroke and recommends a more detailed examination by a neurologist.

Sequential Descriptive AI

The ophthalmologist then shares his interpretation of the fundus photo with you.

He then informs you that he will now use a computer program based on artificial intelligence (AI) to obtain an additional evaluation of the fundus photo. This AI program measures the characteristics of the retina, such as the thickness of the blood vessels, and marks certain features that may indicate whether or not there is an increased risk of disease.

However, the AI program is not used to suggest a diagnosis to the doctor, but serves purely as visual support in evaluating the photo.

The doctor informs you of the results of the AI program and describes whether they correspond to his own assessment and how they are incorporated into the overall evaluation.

He informs you that you have an increased risk of stroke and recommends a more detailed examination by a neurologist.

Concurrent Diagnostic AI

The ophthalmologist informs you that he is using a computer program based on artificial intelligence (AI) for visual and diagnostic support in evaluating the fundus photo. This AI program measures the characteristics of the retina, such as the thickness of the blood vessels, and marks certain features that may indicate whether or not there is an increased risk of disease.

The AI program also suggests a preliminary diagnosis to the doctor. The AI program thus provides the doctor with both visual support in evaluating the photo and assistance in making the diagnosis.

The doctor then summarizes the findings for you.

He informs you that you have an increased risk of stroke and recommends a more detailed examination by a neurologist.

Sequential Diagnostic AI

The ophthalmologist then shares his interpretation of the fundus photo with you.

He then informs you that he will now use a computer program based on artificial intelligence (AI) to obtain an additional evaluation of the fundus photo. This AI program measures the characteristics of the retina, such as the thickness of the blood vessels, and marks certain features that may indicate whether or not there is an increased risk of disease.

The AI program also suggests a preliminary diagnosis to the doctor. The AI program thus provides the doctor with both visual support in evaluating the photo and assistance in making the diagnosis.

The doctor informs you of the results of the AI program and describes whether they correspond with his own assessment and how they are incorporated into the overall evaluation.

He informs you that you have an increased risk of stroke and recommends a more detailed examination by a neurologist.

***Follow-up***

**Please now imagine the following situation:**

Two years ago, you were diagnosed with glaucoma affecting your left eye. This is an eye disease that can be caused by abnormal intraocular pressure, for example, and permanently damages the optic nerve. It causes "blind spots" in your vision. If the disease progresses, the affected eye can go blind. Medicated eye drops can often prevent the disease from worsening.

Now it's time for one of your regular check-ups. Since your regular eye doctor is on vacation for an extended period, you visit a substitute practice. Once there, you are asked to take a seat in the waiting area.

After a while, a medical assistant calls you into a consultation room and takes various measurements, including intraocular pressure and visual field measurements (perimetry).

She also takes a photo of your retina using a fundus camera, which can provide information about possible changes in your glaucoma. Fundus photos can be used, for example, to detect whether certain retinal structures, such as the optic nerve head, have undergone noticeable changes.

After a short wait, the ophthalmologist enters the consultation room. He asks you if you have any symptoms and if you are taking your medication regularly. He then briefly reviews the results of the prior examination and pulls up the fundus photo of your retina on his computer. He examines it and compares the current images with previous ones.

No AI

The ophthalmologist then summarizes the results of the examinations for you and shares his interpretation of the fundus photo.

He informs you that despite the medication, the glaucoma has worsened and your "blind spots" have enlarged. The doctor recommends that you undergo laser treatment.

Concurrent Descriptive AI

The ophthalmologist informs you that he is using a computer program based on artificial intelligence (AI) as a visual aid in evaluating the fundus photograph. This AI program measures certain retinal structures, such as the structure of the optic nerve head. In addition, it creates a trend by comparing the current images with previous images.

However, the AI program is not used to suggest a diagnosis to the doctor, but serves purely as visual support in evaluating the photo.

The doctor then summarizes all the findings for you.

He informs you that, despite the medication, the glaucoma has worsened and your "blind spots" have enlarged. The doctor recommends that you undergo laser treatment.

Sequential Descriptive AI

The ophthalmologist then summarizes the results of the examinations for you and shares his interpretation of the fundus photo.

He then informs you that he will now use a computer program based on artificial intelligence (AI) to obtain an additional evaluation of the fundus photo. This AI program measures certain retinal structures, such as the structure of the optic nerve head. In addition, it creates a trend by comparing the current images with previous images.

However, the AI program is not used to suggest a diagnosis to the doctor, but serves purely as visual support in evaluating the photo.

The doctor informs you of the results of the AI program and describes whether they correspond to his own assessment and how they are incorporated into the overall evaluation.

He informs you that, despite the medication, the glaucoma has worsened and your "blind spots" have enlarged. The doctor recommends that you undergo laser treatment.

Concurrent Diagnostic AI

The ophthalmologist informs you that he is using a computer program based on artificial intelligence (AI) for visual and diagnostic support in evaluating the fundus photo. This AI program measures certain retinal structures, such as the structure of the optic nerve head. In addition, it creates a trend by comparing the current images with previous images.

The AI program also suggests a preliminary diagnosis to the doctor. The AI program thus provides the doctor with both visual support in evaluating the photo and assistance in making the diagnosis.

The doctor then summarizes all the findings for you.

He informs you that, despite the medication, the glaucoma has worsened and your "blind spots" have enlarged. The doctor recommends that you undergo laser treatment.

Sequential Diagnostic AI

The ophthalmologist then summarizes the results of the examinations for you and shares his interpretation of the fundus photo.

He then informs you that he will now use a computer program based on artificial intelligence (AI) to obtain an additional evaluation of the fundus photo. This AI program measures certain retinal structures, such as the structure of the optic nerve head. In addition, it creates a trend by comparing the current images with previous images.

The AI program also suggests a preliminary diagnosis to the doctor. The AI program thus provides the doctor with both visual support in evaluating the photo and assistance in making the diagnosis.

The doctor informs you of the results of the AI program and describes whether they correspond with his own assessment and how they are incorporated into the overall evaluation.

He informs you that despite the medication, the glaucoma has worsened and your "blind spots" have enlarged. The doctor recommends that you undergo laser treatment.
