# Supplementary material for: Effects of Type and Timing of Clinician-Facing AI Support on Patient Trust in Medical Consultations: 2 Vignette Experiments
Source: J Med Internet Res. 2026 Aug 21;28:e93172. doi: 10.2196/93172 (PMC13496942; doi:10.2196/93172)
Supplement: Supplementary file 4 [file jmir-v28-e93172-s004.docx]

**Multimedia Appendix 4**

| **Item** | **Scale** |
| --- | --- |
| **Trust in the Medical Decisions** |  |
| I think the correct diagnosis has been made. | 1 = Completely disagree; 7 = Completely agree |
| I think the correct treatment advice has been given. | 1 = Completely disagree; 7 = Completely agree |
| I trust the medical decisions made in this situation. | 1 = Completely disagree; 7 = Completely agree |
|  |  |
| **Perceived Provider Trustworthiness** |  |
| I believe that this medical provider acts in my best interest. | 1 = Completely disagree; 7 = Completely agree |
| If I need help, this medical provider will do their best to help me. | 1 = Completely disagree; 7 = Completely agree |
| This medical provider is interested in my well-being and not just in their own. | 1 = Completely disagree; 7 = Completely agree |
| This medical provider is truthful in their interactions with me. | 1 = Completely disagree; 7 = Completely agree |
| I would describe this medical provider as honest. | 1 = Completely disagree; 7 = Completely agree |
| This medical provider would keep their commitments to me. | 1 = Completely disagree; 7 = Completely agree |
| This medical provider is sincere and authentic. | 1 = Completely disagree; 7 = Completely agree |
| This medical provider is competent and effective in medical matters. | 1 = Completely disagree; 7 = Completely agree |
| This medical provider fulfills their role of providing medical services very well. | 1 = Completely disagree; 7 = Completely agree |
| Overall, this medical provider is a capable and competent provider of medical services. | 1 = Completely disagree; 7 = Completely agree |
| In general, this medical provider is very knowledgeable in medical matters. | 1 = Completely disagree; 7 = Completely agree |
|  |  |
| **Perceived Uniqueness Neglect** |  |
| This medical provider would not recognize the uniqueness of my medical condition. | 1 = Completely disagree; 7 = Completely agree |
| This medical provider would not take my unique circumstances into account. | 1 = Completely disagree; 7 = Completely agree |
| This medical provider would not tailor the treatment recommendation to my individual case. | 1 = Completely disagree; 7 = Completely agree |
|  |  |
| **Willingness to Follow the Advice** |  |
| How likely is it that you would follow the medical advice? | 1 = Not likely at all; 7 = Very likely |
|  |  |

*Note.* Items were back-translated from English to German.
