# Supplementary material for: Effects of Type and Timing of Clinician-Facing AI Support on Patient Trust in Medical Consultations: 2 Vignette Experiments
Source: J Med Internet Res. 2026 Aug 21;28:e93172. doi: 10.2196/93172 (PMC13496942; doi:10.2196/93172)
Supplement: Supplementary file 5 [file jmir-v28-e93172-s005.docx]

**Multimedia Appendix 5**

**Additional information on the use of ChatGPT-4 in the qualitative analysis workflow**

**Technical details**

ChatGPT-4 (OpenAI) was accessed via the web interface in spring 2025. The language of prompting and data was German. All prompts were used in one conversation. Qualitative free-text responses from Study 1 were copied from the survey export and pasted into the ChatGPT-4 web interface as plain text. The web interface accepted the submitted text and returned an output without displaying an input-length error message or truncation warning. However, because the ChatGPT web interface did not provide token counts or detailed context-handling logs, we cannot retrospectively verify the exact number of tokens processed or fully exclude context loss. As described in the main text, human coders subsequently reviewed all candidate themes against the original free-text responses, and the final coding scheme, interpretation, and reported theme frequencies were based on human coding of the raw responses rather than on model-generated classifications or counts.

**Q1 prompt (German):**

In einer online Studie sollten sich die Versuchspersonen verschiedene Situationen vorstellen, in denen sie aufgrund bestimmter Symptome einen Augenarzt aufsuchen. Dort werden sie untersucht und es werden Bilder ihrer Netzhaut aufgenommen. Je nach experimenteller Bedingung werden diese Bilder von einem Arzt oder einem Arzt mit KI-Unterstützung ausgewertet. Anschließend stellt der Arzt eine Diagnose und gibt eine Handlungsempfehlung. Am Ende der Studie wurden die Versuchspersonen gebeten, in einem Freitextfeld zu beschreiben, welche Überlegungen eine Rolle gespielt haben, während sie Fragen zu Vertrauen in Diagnosen oder zur Vertrauenswürdigkeit des medizinischen Dienstleisters oder zur Absicht, der medizinischen Handlungsempfehlung zu folgen, beantwortet haben. Bitte erstelle eine Liste von sich wiederholenden Thematiken in den Antworten der Versuchspersonen.

**Q1 LLM-suggested themes (German):**

1. Vertrauen und Arzt-Patienten-Beziehung

2. Einstellung zur Künstlichen Intelligenz (KI)

3. Merkmale der medizinischen Handlung

4. Systemisches Vertrauen & institutionelle Faktoren

5. Rezeption der Studie / Methodisches

**Q2 prompt (German):**

Erstelle nun eine Liste an sich wiederholenden Thematiken in den folgenden Antworten der Versuchspersonen auf die Frage, wie sie den Einsatz von KI in den beschriebenen Situationen fanden oder finden würden.

**Q2 LLM-suggested themes (German):**

1. KI als unterstützendes Werkzeug

2. Misstrauen und Unsicherheit gegenüber KI

3. Bedeutung der ärztlichen Expertise

4. Potenzial zur Fehlervermeidung und Genauigkeit

5. Zeitersparnis und Effizienz

6. Wunsch nach Transparenz und Kontrolle

7. Sorge um Vertrauensverlust

8. Individuelle Besonderheiten vs. Standardisierung

**Q3 prompt (German):**

Erstelle nun eine Liste an sich wiederholenden Thematiken in den folgenden Antworten der Versuchspersonen auf die Frage, was ihr Vertrauen in die Diagnose / Handlungsempfehlung erhöhen würde.

**Q3 LLM-suggested themes (German):**

1. Ärztliche Kommunikation & Aufklärung

2. Zweitmeinungen & ärztliche Bestätigung

3. Beziehung zum Arzt / Vertrauen

4. Validierung & Qualitätssicherung

5. Zusätzliche oder alternative Untersuchungen

6. Verfahren & Abläufe

7. Wissen und Kompetenz

8. Vertrauensbildende Maßnahmen

**Q1 prompt (English):**

In an online study, participants were asked to imagine various situations in which they visit an ophthalmologist because of certain symptoms. There, they are examined and images of their retina are taken. Depending on the experimental condition, these images are evaluated by a physician or by a physician with AI support. The physician then makes a diagnosis and gives a recommendation for action. At the end of the study, participants were asked to describe in a free-text field what considerations played a role while they were answering questions about trust in diagnoses, the trustworthiness of the medical provider, or the intention to follow the medical recommendation for action. Please create a list of recurring themes in the participants’ responses.

**Q1 LLM-suggested themes (English):**

1. Trust and the doctor–patient relationship

2. Attitudes toward Artificial Intelligence (AI)

3. Characteristics of the medical intervention/action

4. Systemic trust & institutional factors

5. Perception of the study / methodological aspects

**Q2 prompt (English):**

Now create a list of recurring themes in the following participants’ responses to the question of how they found or would find the use of AI in the described situations.

**Q2 LLM-suggested themes (English):**

1. AI as a supportive tool

2. Distrust and uncertainty toward AI

3. Importance of medical expertise

4. Potential for error reduction and accuracy

5. Time savings and efficiency

6. Desire for transparency and control

7. Concern about loss of trust

8. Individual differences vs. standardization

**Q3 prompt (English):**

Now create a list of recurring themes in the following participants’ responses to the question of what would increase their trust in the diagnosis / recommendation for action.

**Q3 LLM-suggested themes (English):**

1. Physician communication & patient education

2. Second opinions & medical confirmation

3. Relationship with the physician / trust

4. Validation & quality assurance

5. Additional or alternative examinations

6. Procedures & processes

7. Knowledge and competence

8. Trust-building measures
