# Supplementary material for: Effects of Type and Timing of Clinician-Facing AI Support on Patient Trust in Medical Consultations: 2 Vignette Experiments
Source: J Med Internet Res. 2026 Aug 21;28:e93172. doi: 10.2196/93172 (PMC13496942; doi:10.2196/93172)
Supplement: Supplementary file 6 [file jmir-v28-e93172-s006.docx]

**Checklist form - Guideline for RepOrting Vignette Experiments (GROVE)***

Note: this document was originally published in the journal ‘Patient Education and Counseling’ under Creative Commons Attribution 4.0 International License (see <http://creativecommons.org/licenses/by/4.0/>) as Appendix 1 to the following article: Hillen, M.A., Visser, N.C., Labrie, N.H.M. et al. Development of GROVE: a Guideline for RepOrting Vignette Experiments conducted in a healthcare context. Pat Educ Couns (2025). DOI: <https://doi.org/10.1016/j.pec.2025.108750>

| **Criterion** | **Description** | **Location in manuscript where item is reported** | **Details on methodological approach**** |
| --- | --- | --- | --- |
| **1. Rationale** | Provide a rationale for the use of an experimental vignette-based design, including an explanation why the study could not be conducted in a non-simulated setting. | **Section 2. Studies Overview** |  |
| **2. Vignette content** | Describe in detail how the vignette content was developed and refined, and explain any choices made. |  | *Report all relevant information below (sub-criteria 2.1-2.5)* |
| ***2.1. Clinical scenario*** | Describe and explain in detail how the healthcare scenario was developed and what it entailed. Include information about the sources used to inform vignette content, key characteristics of the portrayed characters, and the setting described in the vignette. | **Sections 2. Studies Overview, 4.1.3. Vignettes, and 5.1.2. Design and Procedure, Vignettes, and Measures** | In the German vignettes, the male form of “physician” was used in all vignettes, as the majority of consultants in ophthalmology in Germany is male (Englmaier et al., 2025). We decided against introducing gender as an additional manipulation, as this would substantially increase the necessary sample size. |
| ***2.2. Manipulation &   standardization*** | Describe what the experimental manipulations are (i.e., operationalization of the phenomenon under study), detailing which elements of the scenario were varied and how. Also report how other elements in the vignette were kept constant and provide information on vignette duration or length. | **Sections 4.1.1. Participants, 4.1.3. Vignettes, 5.1.1. Participants, 5.1.2. Design and Procedure, Vignettes, and Measures, and Supplementary Materials** | No additions to the manuscript. |
| ***2.3. Mode of delivery*** | Describe and explain the delivery modality and provide any information necessary for replication. Explain choices regarding narrative perspective and amount of detail described. Describe how participants were introduced to the vignette and in which setting data were collected. | **Sections 2. Studies Overview and 4.1.3. Vignettes** | The online format enabled nation-wide participation and efficient recruitment of large samples necessary to compare multiple experimental conditions while ensuring standardized presentation of the clinical scenarios. To maintain data quality despite remote participation, multiple attention checks, manipulation checks, and predefined exclusion criteria were implemented. |
| ***2.4. Expert   involvement*** | Explain who were involved in developing the vignettes, highlighting their particular expertise and contributions. | **Sections 2. Studies Overview, and 4.1.3. Vignettes** | No additions to the manuscript. |
| ***2.5. Pilot testing*** | Describe if, how, and when pilot testing was used in the vignette development process. Explain whether and how this affected the vignette content and format. | **Section 2. Studies Overview** | Participants for the pilot study were sampled from two subject pools also used for the main data collection and therefore have similar demographic characteristics. The pilot study included the same outcome measures used in the main studies as well as one additional item, which was removed from the main studies as it was not a primary outcome and to avoid overly long participation durations (the extension of vignettes in the main studies was prioritized regarding time spent during participation). |
| **3. Outcomes &   participant   instructions** | Explain the selected study outcome(s) for the vignette study, particularly how these outcome(s) relate to real-world outcomes of interest. | **Section 4.1.4. Measures** | No additions to the manuscript. |
| **4. Vignette validity &   realism** | Report how manipulation success of the independent variable(s) of interest was evaluated (i.e., manipulation check). Also describe if and how realism and aspects of participant engagement with the scenario were assessed. | **Sections 4.1.2. Design and Procedure, 4.1.3. Vignettes, 5.1.2. Design and Procedure, Vignettes, and Measures, and Supplementary Materials** | No additions to the manuscript. |
| **5. Participants** | Provide a rationale for the choice of study participants (e.g., analogue patients), both in relation to the target population and to the characters portrayed in the vignettes. | **Sections 4.1.1. Participants, 4.2.1. Sample Demographics, 5.1.1. Participants, and 5.2.1. Sample Demographics** | The study focused on analogue patients to examine how laypersons who may encounter AI-supported care perceive hybrid clinical decision-making. Although the sample was skewed toward younger adults, this approach enabled recruitment of the large sample sizes required to compare multiple experimental conditions with adequate statistical power, which would have been difficult to achieve with clinical patient populations. In addition, the selected scenarios included both acute and preventive ophthalmic contexts relevant across age groups. Engagement with the vignettes was encouraged through first-person narratives and contextual images illustrating the clinical setting. |
| **6. Accessibility** | Include information on the availability of the final vignettes and pilot data for research, teaching, or commercial purposes. Detail any restrictions to access and (re)use of the vignettes and data. | **Supplementary Materials** | Licensed under CC BY 4.0. |

*The order of reporting these criteria is intended to be flexible. Information can be combined or reorganized and information may be placed in any manuscript section, figure, table and or supplementary material, depending on the study content and journal requirements.

**Authors may report additional methodological details in this column beyond the information included in their main manuscript.
